# Mechanisms of Alignment in Feeding Aphids on the Plant Stem

**DOI:** 10.1002/ece3.70799

**Published:** 2025-01-14

**Authors:** Ayumi Kudo

**Affiliations:** ^1^ Department of Biology, Graduate School of Sciences and Technology for Innovation Yamaguchi University Yamaguchi Japan

**Keywords:** body axis, body position syndrome, phloem‐sap feeder, sap‐sucking insects

## Abstract

Aphids are observed on various plant species, with most aphids feeding downward on stems. In this study, I studied the variations in feeding postures of aphids and their mechanisms. My field observations revealed that the majority of individuals from most species fed facing downward, or more precisely, towards the roots. Exceptions included *Indomegoura indica* on the scapes of *Hemerocallis* spp., which were aligned with their head facing the sky. Next, I investigated how plant orientation affects three aphid species, *Macrosiphoniella yomogifoliae*, *Megoura crassicauda* and *I. indica* with different body alignments on upright immature stems. On the stems of the inverted plants, the number of *Ma. yomogifoliae* and *Me. crassicauda* in the upward position (head facing the root of the plant) was significantly greater than that in the downward position (head facing the shoot apex of the plant). If their posture is affected by gravity or by certain advantages of the headstand posture, *Ma. yomogifoliae* and *Me. crassicauda* are expected to align in a consistent direction, regardless of the orientation of the plant. This suggests that plant cues influence their posture. In contrast, the majority of *I. indica* were aligned with the head facing the sky on the scape, regardless of *Hemerocallis*'s direction. This result indicates that the feeding posture of *I. indica* is affected by gravity and/or aphid's intrinsic factors. Therefore, this study provides a new perspective on the factors influencing aphid feeding posture preferences.

## Graphical abstract

This study provides the first evidence of a common feeding posture among most aphid species affected by plant cues, rather than gravity and other intrinsic factors. Also, I gained a unique insight into *Indomegoura indica* as they were exceptionally aligned with the head towards the sky on the stem of the upward plant and did not share the preference for orientation on the stem with the majority of aphid species.

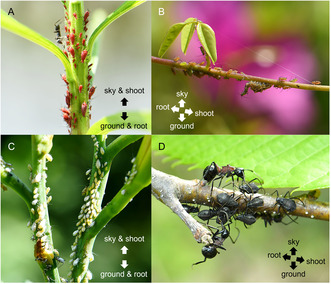

## 1 Introduction

**FIGURE 1:**
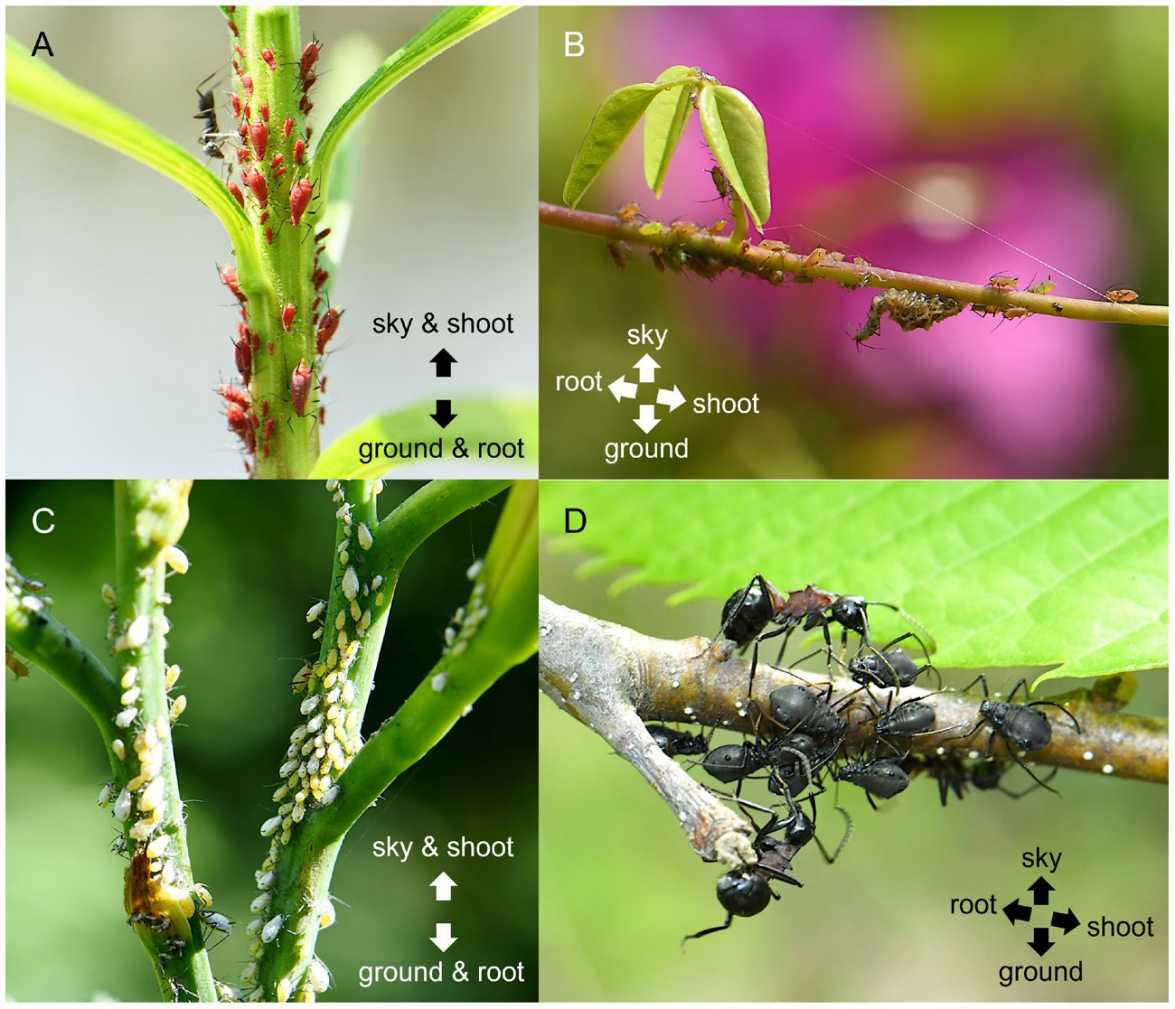
(A) Headstand posture of *Uroleucon nigrotuberculatum* on the stem of *Solidago* sp. (B) The majority of *Sitobion akebiae* positioned with the head towards the root on the horizontal vein of *Akebia trifoliata* to the ground. (C) The majority of *Indomegoura indica* were aligned with the head towards the sky on the scape of *Hemerocallis* sp. (D) Low bias in body orientation of *Lachnus tropicalis* on the twig of *Castanea crenata* . Arrows indicate the vertical direction and vertical axes of plants. All photographs were taken by the author in Yamaguchi, Japan. The orientation and number of aphids on each plant are shown in Table 1 and Appendix S2.

Aphids suck the phloem sap from various plant species. During both day and night, most aphids fold and align face‐down on plant stems (e.g., *Uroleucon nigrotuberculatum* on the stem of *Solidago* sp. in Figure 1A). The headstand posture is common in most aphid species; however, there are few reports, and they lack detailed descriptions (Dixon 1958; Pollard 1973; Klingauf 1987; Lazzari and Zonta‐de‐Carvalho 2012). The biological significance of headstand posture has been discussed, with suggestions that this behaviour may facilitate rostral bending and stylet insertion (Pollard 1973) and enhance predator detection efficiency (Dixon 1958); however, this phenomenon has not been empirically tested. Furthermore, the mechanism driving the body orientation remains unknown. Three potential orientation factors have been proposed: (i) gravity, (ii) plant cues and (iii) aphid's intrinsic factors (Pollard 1973). Gravity is one of the most ubiquitous and least fluctuating environmental sensory cues. As with most living systems, aphids sense gravity and often exhibit positive geotaxis after landing (Pettersson, Tjallingii, and Hardie 2007). Second, external plant cues (i.e., chemical and physical cues on the plant surface) and/or internal plant cues (i.e., phloem sap flow direction) can influence aphid behaviour. Third, aphid's intrinsic factor seems to give them some advantages in operating their mouthparts and avoiding climbing predators, such as coccinellids (Dixon 1958; Pollard 1973). If any plant–aphid combination shares the aphid's headstand posture regardless of the situation, the aphid's body orientation will likely be affected by gravity rather than a characteristic trait of certain species. In this study, I assessed the consistency of feeding postures among different aphid species based on field observations and investigated how the orientation of plants affected aphids. Investigating the factors influencing the feeding posture of aphids may help identify new stimuli involved in the selection and determination of feeding sites. This finding is expected to promote further empirical research on aphid–plant interactions and contribute to a broader understanding of host specialisation and adaptability in aphids.

## 2 Materials and Methods

### 2.1 Field Observations

Field observations were conducted in Japan between April and June 2022, in July 2023 and in March, October and November 2024. All photographs of the aphids surrounding the plant stem were taken from a single angle in the field to count aphids that were not easily identifiable in the field. The number of aphids in both the upward and downward positions on the plant stems was recorded from the photographs.

### 2.2 Evaluation of Feeding Posture in Response to Plant Orientation in the Laboratory

To compare the preferences and factors influencing feeding posture, I selected the combinations of *Artemisia indica* with *Macrosiphoniella yomogifoliae*, *Vicia faba* with *Megoura crassicauda* and *Hemerocallis* spp. with *Indomegoura indica. Ma. yomogifoliae* and *Me. crassicauda* represent the typical type that feeds in a headstand posture, whereas *I. indica* is an exceptional type that feeds in an upward position on the plant stem in the field. These aphids were selected based on their contrasting feeding postures.

#### 2.2.1 Macrosiphoniella yomogifoliae

*Ma. yomogifoliae* were originally collected from *A. indica* on 19, 20 and 24 October 2024, and the population was maintained on *A. indica* under laboratory conditions. *A. indica* for the experiments was placed in pots (85 or 120 mm in height and, 110 or 150 mm upper diameter), maintained under natural conditions and transferred to the laboratory prior to the commencement of the experiment. To prevent the soil and plants from falling out when the pot was inverted, the opening of the pot was covered with plastic wrap. This supports the soil and plants, thereby preventing their descent.

#### 2.2.2 Megoura crassicauda

*Me. crassicauda* was originally collected from *Vicia sativa* on 6 May 2021 and the population was maintained on bean seedlings in the laboratory. Experimental *V. faba* seedlings were grown individually in plastic cups (28 mm in height, 68 mm upper diameter) with moist cotton. The centre of each lid was cut with a 1‐cm diameter hole through which the stem could be threaded.

#### 2.2.3 Indomegoura indica

*I. indica* was originally collected from *Hemerocallis fulva* on 16 July 2023 and the population was maintained on *Hemerocallis* spp. under laboratory conditions. *Hemerocallis* spp. with flower buds for the experiments were placed in pots (120 or 150 mm in height, 150 or 190 mm upper diameter), maintained under natural conditions and transferred to the laboratory prior to the commencement of the experiment. The same approach used for *A. indica* was applied to prevent soil and plant dislodgement.

Older instars and adult aphids were selected from the colonies reared in the laboratory and examined. Twenty aphids were gently transferred to the stems of inverted host plants using a brush. I conducted control experiments with upright host plants to examine whether laboratory conditions themselves affected the feeding posture of the three aphid species. After more than 10 h of acclimatisation to the plant, the number of aphids in the upward and downward positions on the stem was counted. As the test plants were not isolated (e.g., with nets) and all laboratory experiments were conducted in the same room; therefore, it is possible that some aphids may have migrated between the focal test host plant and other host plants. Aphids were excluded from the dataset if they walked on the stems during observation. All experiments were conducted at a room temperature of 23°C under a 14L:10D photoperiod.

All analyses were conducted using R (version 4.2.3: R Core Team 2023) and R Studio (version 2023.06.0: Posit Team 2023). To assess consistency across the different test plants of the same treatment, the Cochran–Mantel–Haenszel test was used to pool the frequencies of aphid orientation and Tarone's test was used to examine heterogeneity with a null probability of 0.5 (the expected value when orientations were determined randomly) using the R *metafor* package with BH correction.

## 3 Results and Discussion

### 3.1 Feeding Posture in the Field

The prevailing orientation of most aphid species was downward on the plant stems (Table 1, Appendix S2). However, this finding requires further clarification. In the case of leaning stems or, in extreme scenarios, the horizontal vein, the majority of aphids were aligned parallel to the longitudinal axis of the plant body. Rather than positioning their heads towards the ground, they were oriented towards the root of the plant (Figure 1B, Table 1, Appendix S2). Thus, aphids likely prefer a posture facing the roots to a headstand posture, suggesting that the body orientation of aphids is affected by plant cues rather than gravity. In addition, I discovered two interesting findings. First, I found that the majority of *I. indica* aphids were aligned with the head towards the sky on the upward flower stems (scapes) of daylily *Hemerocallis* spp. (Figure 1C, Table 1, Appendix S2). A comparison of *I. indica* in the upward position with other aphid species in the downward position showed that their feeding postures seem to be affected by different factors. Second, in the twigs of the Japanese chestnut, *Castanea crenata* , there was a low bias in the body orientation of *Lachnus tropicalis* aphids (Figure 1D, Table 1, Appendix S2). Likewise, some aphid species belonging to Aphidinae, Hormaphidinae and Lachninae, which feed on mature stems (twigs, branches and trunks) in which secondary growth progressed, such as *Pterocomma pilosum*, *Nipponaphis machilicola* and *Cinara crenata*, also seem to have a low bias in body orientation (see figures in Albrecht 2017; Choi et al. 2018; Moritsu 1983). Most aphid species that feed on mature stems of trees do not alternate hosts and show a lack of preference for feeding posture. It is possible that the shorter stylets of aphid species that feed on immature plant parts, compared to those that feed on mature plant parts, are associated with a preference for headstand feeding posture. Both nymphs and adults of species that feed on tree branches and trunks tend to have longer mouthparts to reach a deeper phloem tissue compared to those that feed on immature plant parts (Lazzari and Zonta‐de‐Carvalho 2012; Chen et al. 2016). Furthermore, adult *Edessa meditabunda* (Heteroptera: Pentatomidae), which feeds on xylem and has shorter stylets, shows a preference for a downward posture on soybean stems (Panizzi and Machado‐Neto 1992). These findings suggest a potential relationship between short stylet and preference for headstand feeding posture, although the underlying mechanism remains unclear.

**TABLE 1:** Aphid orientation on the plant stems in the field.

Aphid	Plant	Maturity of feeding site^a^	Rep.	N of aphids with head towards root	N of aphids with head towards shoot	Proportion of aphids with head towards root	Date of observation	Picture ID^b^
*Megoura crassicauda*	*Vicia sativa*	Immature	1	27	0	1.00	2022/4/20	1
			2	34	2	0.94	2022/4/20	2
			3	26	0	1.00	2022/4/20	3
*Sitobion akebiae*	*Akebia trifoliata*	Immature	1	22	1	0.96	2022/4/20	4
			2	13	1	0.93	2022/4/20	5
			3	17	5	0.77	2022/4/20	6
			4	11	2	0.85	2022/4/20	7
*Hyperomyzus* sp.	*Sonchus* sp.	Immature	1	77	1	0.99	2022/4/22	8
			2	16	2	0.89	2022/4/22	9
			3	28	0	1.00	2022/4/22	10
*Uroleucon picridis*	*Picris hieracioides*	Immature	1	86	0	1.00	2022/4/22	11
			2	35	6	0.85	2022/4/22	12
*Sitobion ibarae*	*Rosa* sp.	Immature	1	71	3	0.96	2022/4/24	13
			2	41	0	1.00	2022/4/24	14
			3	16	0	1.00	2022/4/24	15
*Uroleucon nigrotuberculatum*	*Solidago* sp.	Immature	1	40	0	1.00	2022/5/8	16
			2	22	0	1.00	2022/5/5	17
			3	20	1	0.95	2022/5/8	18
*Brevicoryne brassicae*	*Brassica juncea*	Immature	1	29	3	0.91	2022/5/25	19
			2	16	3	0.84	2022/5/25	20
			3	42	0	1.00	2022/5/25	21
*Uroleucon giganteum*	*Cirsium* sp.	Immature	1	25	0	1.00	2022/6/25	22
			2	8	0	1.00	2022/6/25	23
			3	8	0	1.00	2022/6/25	24
*Indomegoura indica*	*Hemerocallis* sp.	Immature	1	10	65	0.13	2023/7/2	25
			2	6	54	0.10	2023/7/2	26
			3	14	36	0.28	2023/7/16	27
			4	12	30	0.29	2023/7/16	28
			5	1	11	0.08	2023/7/16	29
*Aphis kurosawai*	*Artemisia indica*	Immature	1	43	2	0.96	2023/7/16	30
			2	26	2	0.93	2023/7/16	31
			3	30	3	0.91	2024/10/20	32
			4	18	3	0.86	2024/10/20	33
			5	13	1	0.93	2024/10/20	34
*Macrosiphoniella yomogifoliae*			1	12	0	1.00	2024/10/20	35
			2	14	0	1.00	2024/10/20	36
			3	18	1	0.95	2024/10/20	37
			4	18	1	0.95	2024/10/20	38
			5	12	0	1.00	2024/10/20	39
*Aphis spiraecola*	*Bidens pilosa*	Immature	1	12	1	0.92	2024/11/7	40
			2	28	3	0.90	2024/11/7	41
			3	81	14	0.85	2024/11/8	42
			4	36	2	0.95	2024/11/8	43
*Lachnus tropicalis*	*Castanea crenata*	Mature	1	12	13	0.48	2024/4/17	44
			2	5	4	0.56	2024/4/19	45
			3	4	7	0.36	2024/4/19	46

^a^ Immature: The aphids were feeding on the young green stem of plant. Mature: The aphids were feeding on the older brown stem of plant.

^b^ Refer to Appendix S2.

### 3.2 Response to Plant Orientation Under Laboratory Conditions

**FIGURE 2:**
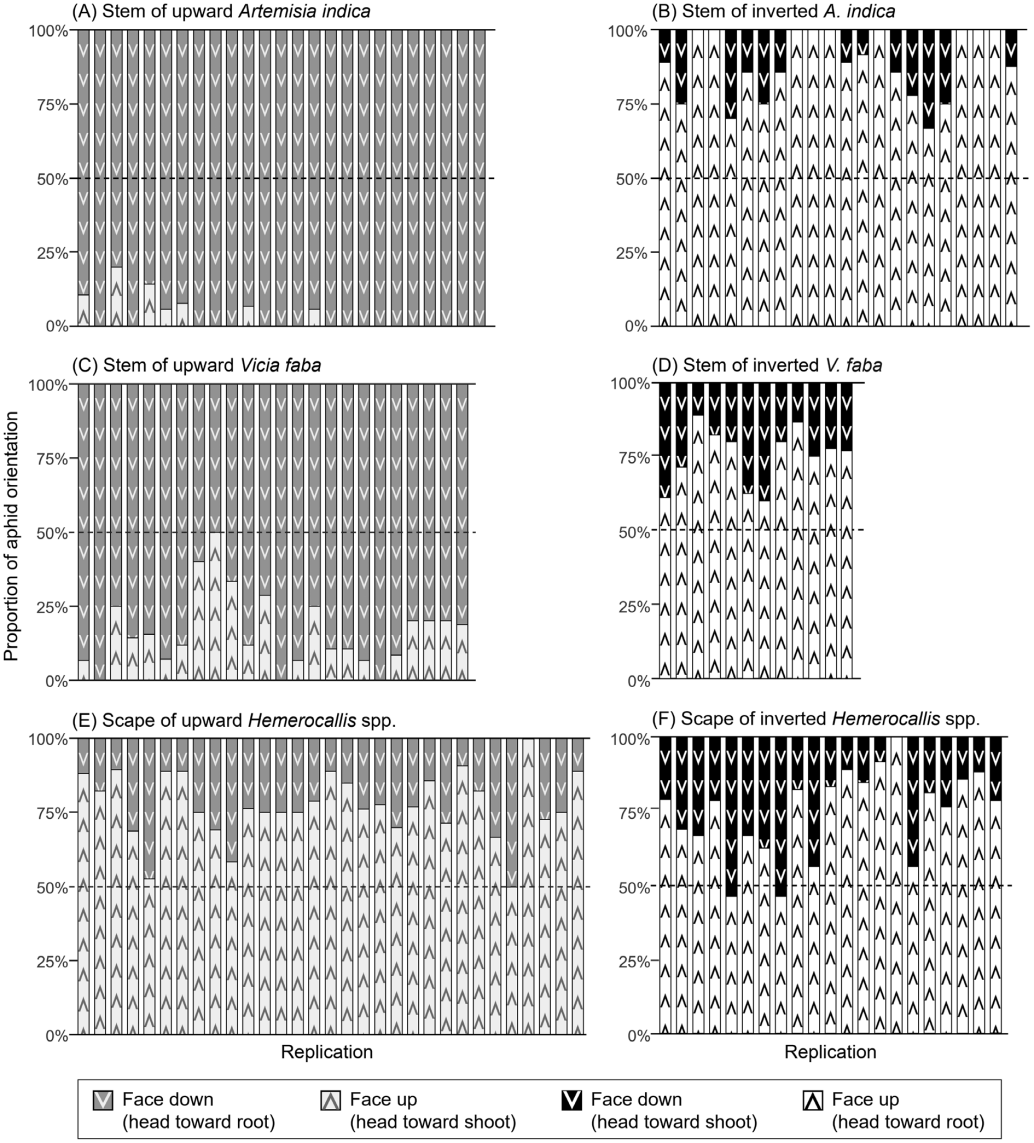
Proportion of aphids with face up and face down in laboratory condition. (A) *Macrosiphoniella yomogifoliae* on the stem of upward *Artemisia indica*. (B) *Ma. yomogifoliae* on the stem of inverted *A. indica* . (C) *Megoura crassicauda* on the stem of upward *Vicia faba* . (D) *Me. crassicauda* on the stem of inverted *V. faba* . (E) *Indomegoura indica* on the scape of upward *Hemerocallis* spp. (F) *I. indica* on the scape of inverted *Hemerocallis* spp.

On the upward‐oriented stems of *A. indica* under laboratory conditions, the number of *Ma. yomogifoliae* in the downward position was significantly higher than that in the upward position (OR = 0.03, 95% confidence interval [CI] = 0.02–0.06, *p* < 0.01, *p* for heterogeneity = 0.87; Figure 2A), as well as in field observations. However, on the stems of inverted *A. indica* , the number of *Ma. yomogifoliae* in the upward position (head towards the root of the plant) was significantly higher than that in the downward position (head towards the shoot apex of the plant) (OR = 7.84, 95% CI = 5.17–11.89, *p* < 0.01, *p* for heterogeneity = 0.56; Figure 2B). A similar trend was observed for *Me. crassicauda*. On the upward‐oriented stem of *V. faba* , *Me. crassicauda* in the downward position exceeded that in the upward position (OR = 0.21, 95% confidence interval [CI] = 0.16–0.29, *p* < 0.01, *p* for heterogeneity = 0.10; Figure 2C). When the *V. faba* was inverted, the abundance of *Me. crassicauda* was significantly greater in the upward position compared to the downward position (OR = 2.80, 95% CI = 1.96–4.01, *p* < 0.01, *p* for heterogeneity = 0.70; Figure 2D). If body orientation were affected only by gravity, aphids would have been oriented towards the ground on the stems of both inverted and upward plants. However, the feeding postures of *Ma. yomogifoliae* and *Me. crassicauda* changed with the plant orientation. Similarly, if their headstand postures offer advantages over operating their mouthparts and avoiding climbing predators such as coccinellids (Dixon 1958; Pollard 1973), they should have been oriented towards the ground on the stem of the inverted plant. However, this was not the case in this study, indicating that the feeding postures of *Ma. yomogifoliae* and *Me. crassicauda* are affected by external and/or internal plant cues, rather than by gravity or aphid's intrinsic factors.

Insects rely on various plant characteristics, including visual, olfactory, gustatory and tactile characteristics, to identify suitable plant species and tissues for foraging. Among these characteristics, hair‐like epidermal structures such as trichomes, which vary in presence, structure and orientation across plant species and organs, appear to influence aphid movement and feeding behaviour (Ni and Qisenberry 1997; reviewed in Guerrieri and Digilio 2008). For example, there were differences in stem hair between the two plants with *Me. crassicauda* feeding on *V. sativa* with short and upturned hairs (8–16 trichomes/mm^2^, average 14 trichomes/mm^2^; 0.05–0.22 mm in length, average 0.10 mm; personal observations by AK) and *V. faba* with no hair. Regardless of these differences in the physical structure of host plants, the majority of *Me. crassicauda* were consistently in the downward position on both host plants. Thus, it is unlikely that external plant cues determine only the posture of the aphid. A plausible internal plant cue is the sap flow direction, as aphids require relatively high turgor pressure to feed efficiently on the phloem (Huberty and Denno 2004; Quandahor et al. 2020). For example, the majority of stink bugs, *Dichelops melacanthus* were in a downward position when they sucked the xylem sap of maize; however, during nonsucking periods, the majority of *D. melacanhus* were in an upward position (Panizzi and Lucini 2019). The authors suggested that the downward position facilitated intake of the xylem sieve, which moved upward from the roots to the shoot apices (Panizzi and Lucini 2019). Unlike stink bugs, most aphids are phloem sap feeders. The phloem sap moves from the source (typically mature leaves) to the sink (e.g., roots, tubers, developing fruits and immature leaves), and the velocity in the phloem shows little diurnal variation (Peuke et al. 2001; Taiz et al. 2015). Therefore, phloem sap flow is likely to influence aphid orientation as it is a less fluctuating and common cue among species. The ratio of aphids in the downward position on the stems of upward plants is usually lower under laboratory conditions than that observed in the field. This observation may also reflect the influence of phloem sap flow on aphid posture, as phloem flow characteristics might change with reduced photosynthetic rates due to the lower light intensity in laboratory conditions compared to outdoor levels. However, the mechanisms underlying the phloem sap transport remain unclear. Our observations call for a better understanding of phloem sap flow in each plant.

To compare the preferences and factors influencing the feeding posture between *I. indica* and most aphid species, I investigated the feeding posture of *I. indica* on upward and downward plants using the same method as that used for *Ma. yomogifoliae* and *Me. crassicauda*. Interestingly, the number of *I. indica* in the upward position was significantly higher than that in the downward position on the scape, regardless of the plant direction (OR = 3.41, 95% CI = 2.76–4.21, *p* < 0.01, *p* for heterogeneity = 0.60 for upward *Hemerocallis* spp. and OR = 2.93, 95% CI = 2.26–3.81, *p* < 0.01, *p* for heterogeneity = 0.10 for downward *Hemerocallis* spp.; Figure 2E,F). This result indicates that, unlike other aphid species, the feeding posture of *I. indica* is affected by gravity and/or the aphid's intrinsic factors, rather than plant cues. Most insects exhibit both positive and negative geotaxis, which may be useful for position control (Jander 1963). Furthermore, head orientation relative to gravity can influence haemolymph flow and air distribution, even in insects with open circulatory systems (Harrison et al. 2020). Thus, in *I. indica* , a unique physiological mechanism may have evolved compared to that in other aphid species.

This study provides the first evidence of a common feeding posture among most aphid species affected by plant cues rather than gravity and other intrinsic factors. In addition, I gained a unique insight into *I. indica* as they were exceptionally aligned with the head facing the sky on the stem of the upward plant and did not share a preference for orientation on the stem with the majority of aphid species. Different morphological traits, such as mouthpart length and body size—which is correlated with mouthpart length (Dixon 1997)—may have evolved in response to the characteristics of the feeding site. Therefore, by examining the morphology of species that exhibit seasonal alternation between different hosts, it may be possible to identify traits that strongly influence feeding posture. Also, the biological differences between *I. indica* and other headstand aphid species that would affect their feeding posture, the costs and benefits of headstand feeding posture, and how these costs and benefits contribute to feeding posture preferences remain unknown and require further study.

## Author Contributions

**Ayumi Kudo:** conceptualization (equal), data curation (equal), formal analysis (equal), investigation (equal), methodology (equal), resources (equal), supervision (equal), visualization (equal), writing – original draft (equal), writing – review and editing (equal).

## Conflicts of Interest

The author declares no conflicts of interest.

## Supporting information


**Appendix S1** Supporting Information
**Table S1**. Number of *Macrosiphoniella yomogifoliae* orientation on the stem of upward *Arutemisia indica* in laboratory condition.
**Table S2**. Number of *Macrosiphoniella yomogifoliae* orientation on the stem of inverted *Arutemisia indica* in laboratory condition.
**Table S3**. Number of *Megoura crassicauda* orientation on the stem of upward *Vicia faba* in laboratory condition.
**Table S4**. Number of *Megoura crassicauda* orientation on the stem of inverted *Vicia faba* in laboratory condition.
**Table S5**. Number of *Indomegoura indica* orientation on the scape of upward *Hemerocallis* spp. in laboratory condition.
**Table S6**. Number of *Indomegoura indica* orientation on the scape of inverted *Hemerocallis* spp. in laboratory condition.

**Appendix S2** Photographs of aphids on plant stems in the field. Warm colour and cool colour points indicate aphids positioned with their heads towards the root and the shoot respectively. All photographs were captured by Kudo (Japan).

## Data Availability

Data are available in Supporting Information (Appendix S1 and S2).
