# Supplementary material for: Mechanisms of Alignment in Feeding Aphids on the Plant Stem
Source: Ecol Evol. 2025 Jan 14;15(1):e70799. doi: 10.1002/ece3.70799 (PMC11732736; doi:10.1002/ece3.70799)
Supplement: Supplementary file 1 — Appendix S1 Supporting Information Table S1. Number of Macrosiphoniella yomogifoliae orientation on the stem of upward Arutemisia indica in laboratory condition. Table S2. Number of Macrosiphoniella yomogifoliae orientation on the stem of inverted Arutemisia indica in laboratory condition. Table S3. Number of Megoura crassicauda orientation on the stem of upward Vicia faba in laboratory condition. Table S4. Number of Megoura crassicauda orientation on the stem of inverted Vicia faba in laboratory condition. Table S5. Number of Indomegoura indica orientation on the scape of upward Hemerocallis spp. in laboratory condition. Table S6. Number of Indomegoura indica orientation on the scape of inverted Hemerocallis spp. in laboratory condition. [file ECE3-15-e70799-s001.pdf]

Table S1. Number of *Macrosiphoniella yomogifoliae* orientation on the stem of upward *Artemisia indica* in laboratory condition.

Rep.	N of aphids with head toward root on stem (face down)	N of aphids with head toward shoot on stem (face up)	N of aphids on leaf	N of walking aphids
1	17	2	0	0
2	3	0	3	0
3	4	1	1	0
4	5	0	11	0
5	12	2	5	0
6	16	1	3	0
7	12	1	6	0
8	20	0	0	0
9	8	0	4	0
10	7	0	1	0
11	14	1	4	0
12	16	0	2	0
13	7	0	0	0
14	8	0	3	0
15	16	1	5	0
16	10	0	3	0
17	20	0	4	0
18	5	0	2	0
19	12	0	0	0
20	14	0	3	0
21	8	0	1	0
22	14	0	6	0
23	10	0	3	0
24	11	0	1	1
25	7	0	1	0

Table S2. Number of *Macrosiphoniella yomogifoliae* orientation on the stem of inverted *Artemisia indica* in laboratory condition.

Rep.	N of aphid with head toward shoot on stem (face down)	N of aphids with head toward root on stem (face up)	N of aphids on leaf	N of walking aphids
1	1	8	4	0
2	1	3	0	0
3	0	10	1	0
4	0	13	1	0
5	3	7	1	0
6	2	12	2	0
7	2	6	1	0
8	2	12	0	0
9	0	9	4	0
10	0	4	9	0
11	0	15	6	0
12	1	8	5	0
13	1	11	5	0
14	0	12	4	0
15	1	6	5	1
16	2	7	3	0
17	5	10	3	1
18	3	9	2	0
19	0	17	4	0
20	0	7	2	1
21	0	3	3	0
22	1	7	2	1

Table S3. Number of *Megoura crassicauda* orientation on the stem of upward *Vicia faba* in laboratory condition.

Rep.	N of aphids with head toward root on stem (face down)	N of aphids with head toward shoot on stem (face up)	N of aphids on leaf	N of walking aphids
1	14	1	4	0
2	7	0	14	0
3	6	2	9	0
4	6	1	10	0
5	11	2	6	0
6	13	1	3	0
7	15	2	3	1
8	9	6	5	0
9	10	10	0	0
10	8	4	7	0
11	15	2	3	0
12	10	4	6	0
13	5	0	15	0
14	14	1	3	1
15	9	3	3	0
16	17	2	4	0
17	17	2	4	0
18	14	1	5	0
19	5	0	0	7
20	11	1	0	0
21	8	2	2	2
22	8	2	3	1
23	16	4	0	0
24	13	3	3	0

Table S4. Number of *Megoura crassicauda* orientation on the stem of inverted *Vicia faba* in laboratory condition.

Rep.	N of aphid with head toward shoot on stem (face down)	N of aphids with head toward root on stem (face up)	N of aphids on leaf	N of walking aphids
1	7	11	0	0
2	2	5	13	0
3	1	8	7	0
4	3	14	3	0
5	1	4	11	0
6	6	10	0	0
7	8	12	0	0
8	3	12	0	0
9	2	13	1	0
10	4	12	1	0
11	4	14	0	0
12	3	10	2	0

Table S5. Number of *Indomegoura indica* orientation on the scape of upward *Hemerocallis* spp. in laboratory condition.

Rep.	N of aphids with head toward root on scape (face down)	N of aphids with head toward shoot on scape (face up)	N of aphids with head toward root on the other parts (face down)	N of aphids with head toward shoot on the other parts (face up)	N of walking aphids
1	2	15	0	1	0
2	3	14	1	0	0
3	2	17	0	0	0
4	5	11	0	0	0
5	9	10	0	0	0
6	2	16	0	2	0
7	2	16	0	0	0
8	4	12	1	2	0
9	4	9	2	3	0
10	10	14	0	0	0
11	4	13	0	0	0
12	5	15	0	0	0
13	5	15	0	0	0
14	5	15	1	2	0
15	4	15	0	1	0
16	2	16	0	2	0
17	3	17	0	2	0
18	5	16	0	0	0
19	4	14	0	3	0
20	6	14	0	0	0
21	3	10	3	2	0
22	3	18	0	0	0
23	4	10	0	7	0
24	1	10	4	7	0
25	3	14	0	0	0
26	2	4	9	3	0
27	4	4	4	10	0
28	0	6	5	2	0
29	3	8	2	5	0
30	2	6	5	5	0
31	1	8	6	5	0

Table S6. Number of *Indomegoura indica* orientation on the scape of inverted *Hemerocallis* spp. in laboratory condition.

Rep.	N of aphids with head toward shoot on scape (face down)	N of aphids with head toward root on scape (face up)	N of aphids with head toward shoot on the other parts (face down)	N of aphids with head toward root on the other parts (face up)	N of walking aphids
1	4	15	0	1	0
2	5	11	0	4	0
3	4	8	3	4	0
4	3	11	1	4	0
5	7	6	0	5	0
6	3	6	0	0	0
7	6	10	0	5	0
8	7	6	1	3	0
9	3	14	0	1	0
10	7	9	2	2	0
11	1	5	0	5	0
12	1	8	1	2	0
13	2	11	1	0	0
14	1	11	0	0	0
15	0	16	1	1	0
16	7	9	1	2	0
17	3	13	1	0	0
18	4	13	0	1	0
19	2	12	0	1	0
20	2	15	2	1	0
21	3	11	1	2	0
