# Supplementary material for: Mechanisms of Alignment in Feeding Aphids on the Plant Stem
Source: Ecol Evol. 2025 Jan 14;15(1):e70799. doi: 10.1002/ece3.70799 (PMC11732736; doi:10.1002/ece3.70799)
Supplement: Supplementary file 2 — Appendix S2 Photographs of aphids on plant stems in the field. Warm colour and cool colour points indicate aphids positioned with their heads towards the root and the shoot respectively. All photographs were captured by Kudo (Japan). [file ECE3-15-e70799-s002.pdf]

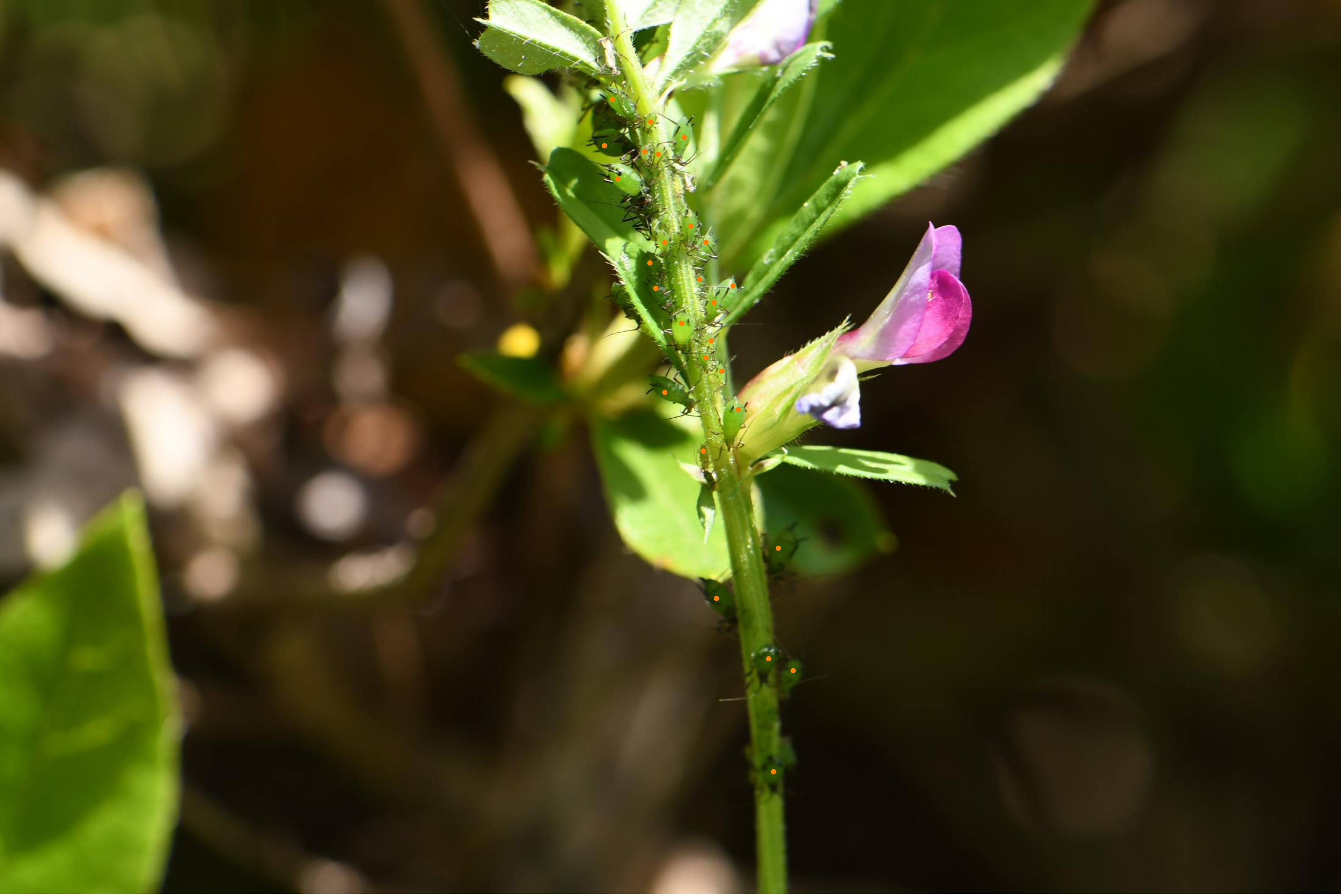

Picture ID: 1, *Megoura crassicauda* on *Vicia sativa*, 20 April 2022.

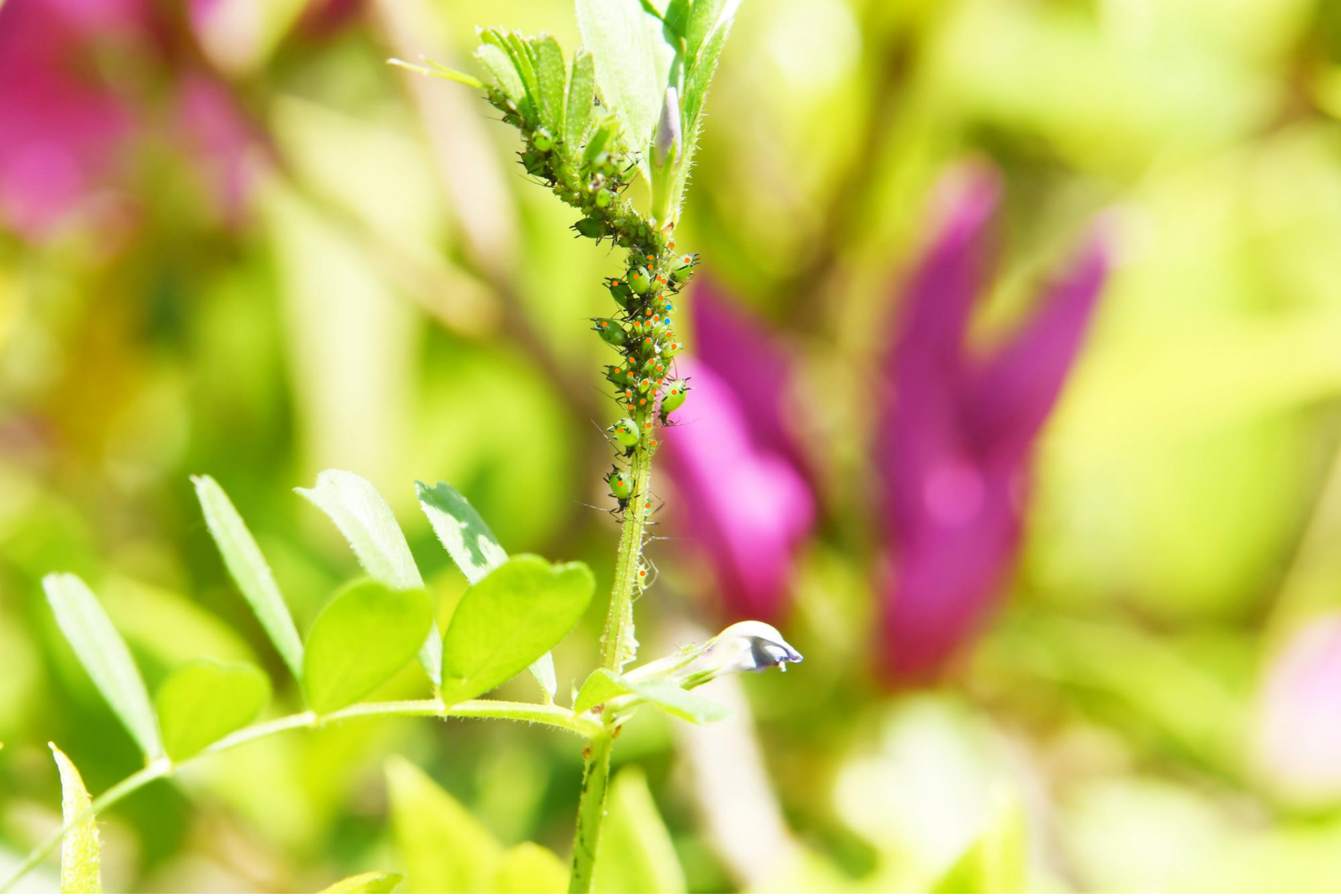

Picture ID: 2, *Me. crassicauda* on *V. sativa*, 20 April 2022.

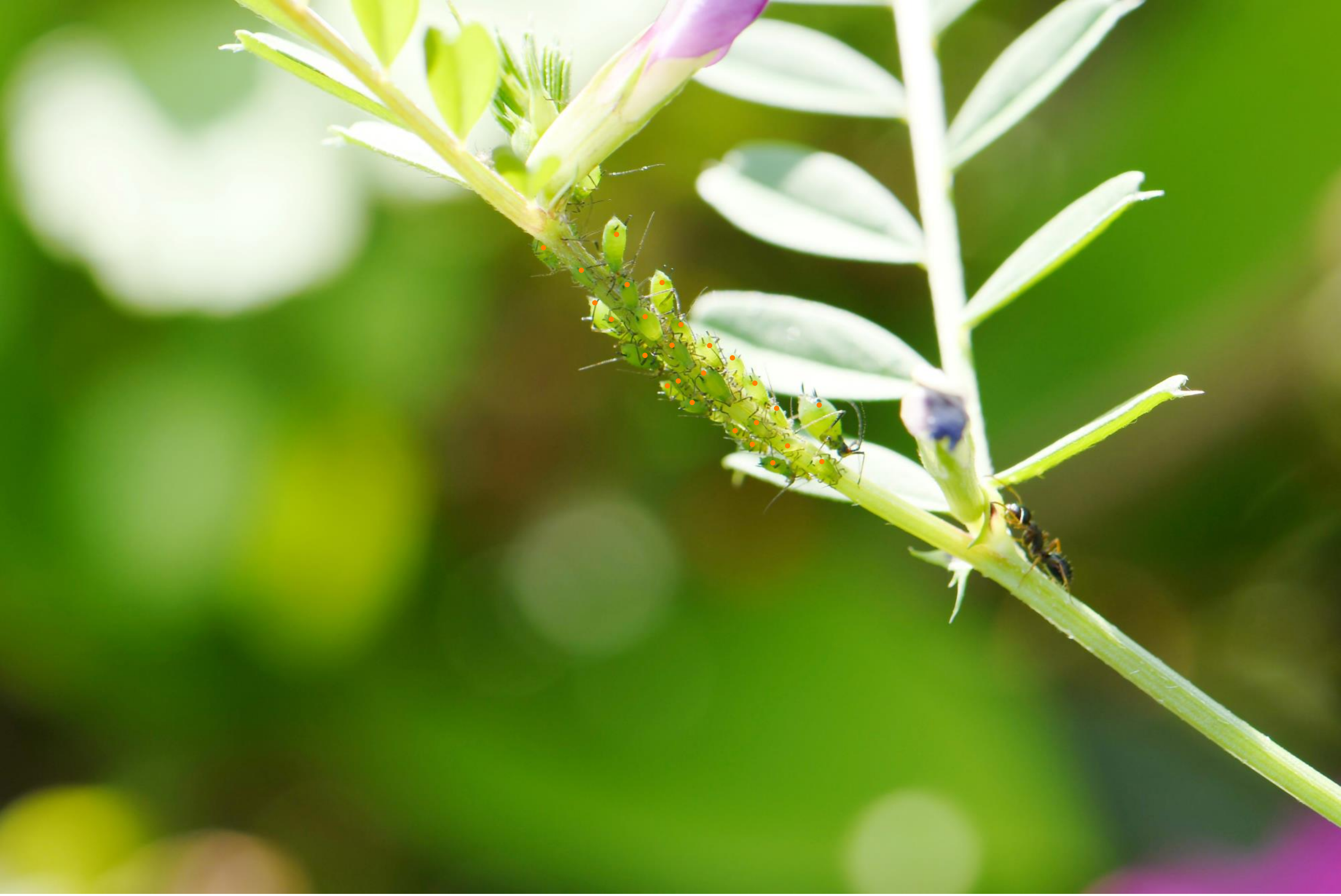

Picture ID: 3, *Me. crassicauda* on *V. sativa*, 20 April 2022.

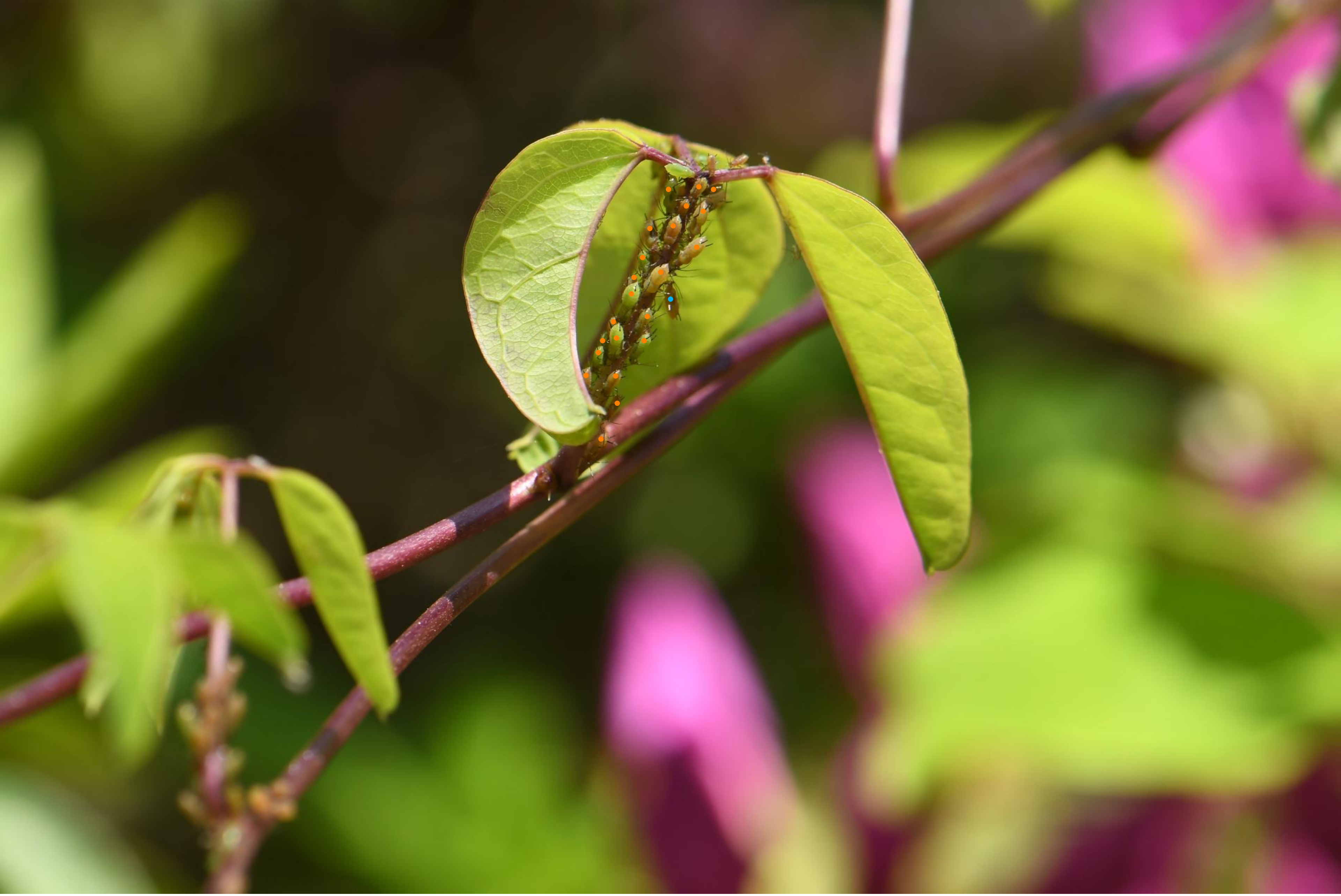

Picture ID: 4, *Sitobion akebiae* on *Akebia trifoliata*, 20 April 2022.

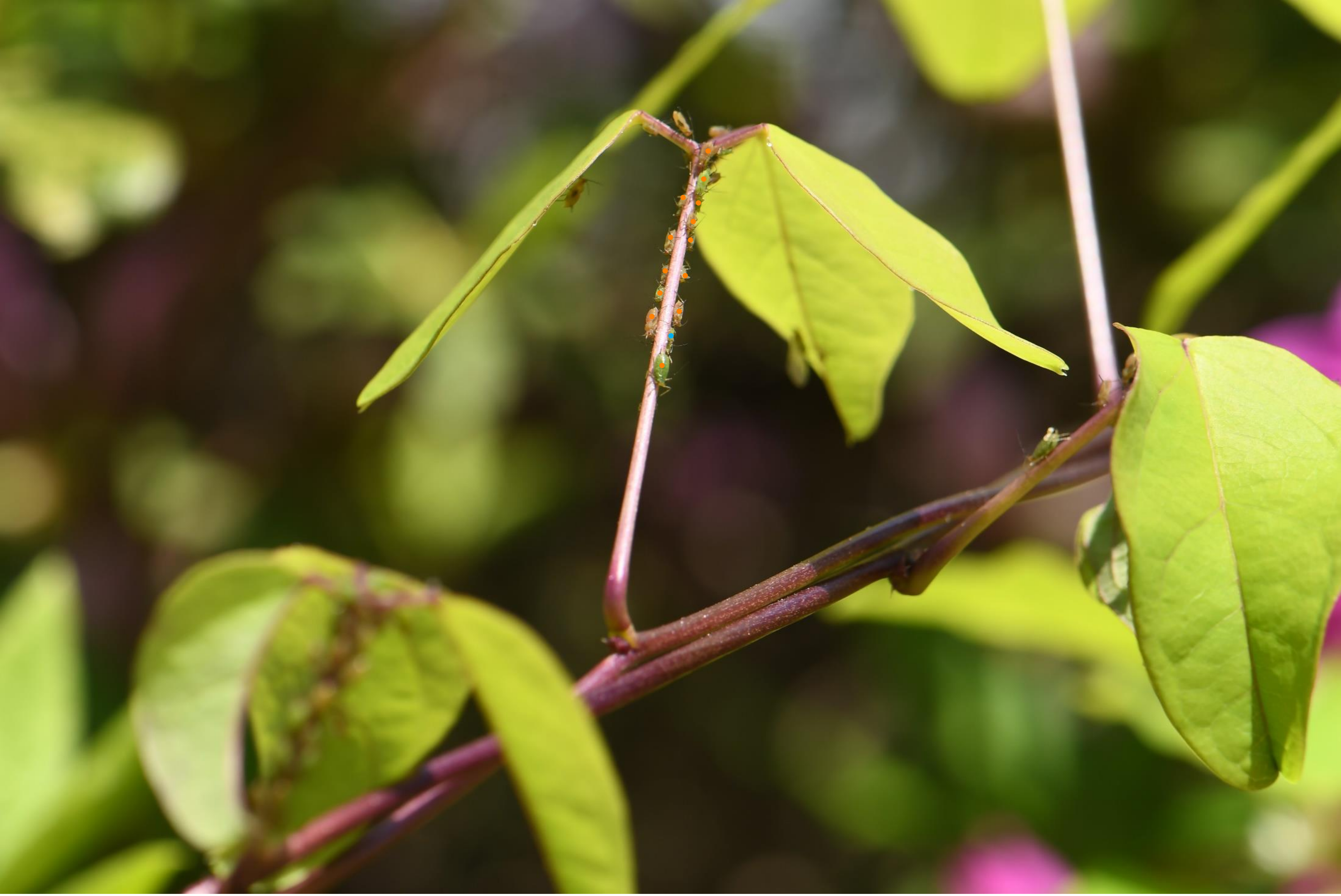

Picture ID: 5, *Si. akebiae* on *Ak. trifoliata*, 20 April 2022.

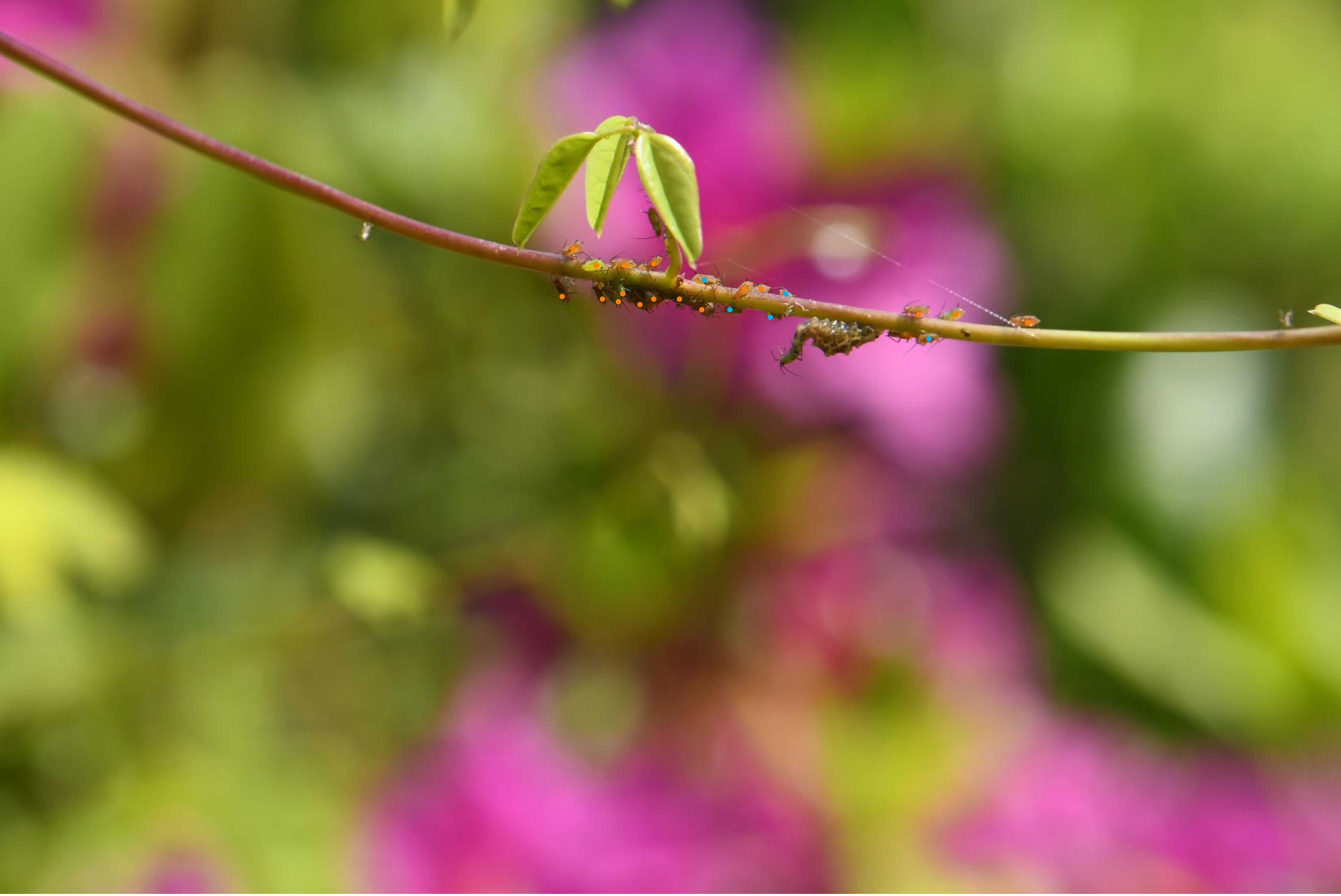

Picture ID: 6, *Si. akebiae* on *Ak. trifoliata*, 20 April 2022.

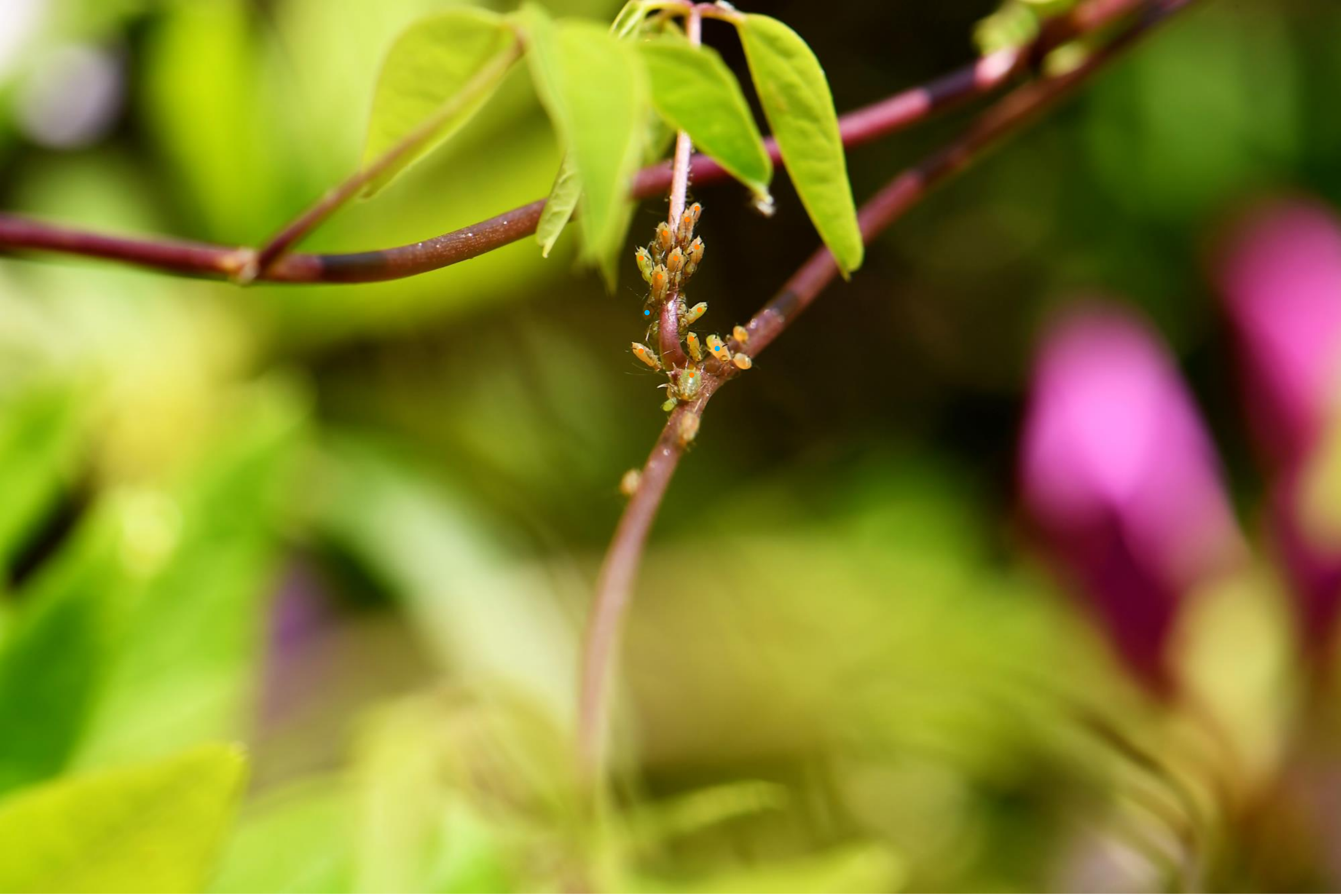

Picture ID: 7, *Si. akebiae* on *Ak. trifoliata*, 20 April 2022.

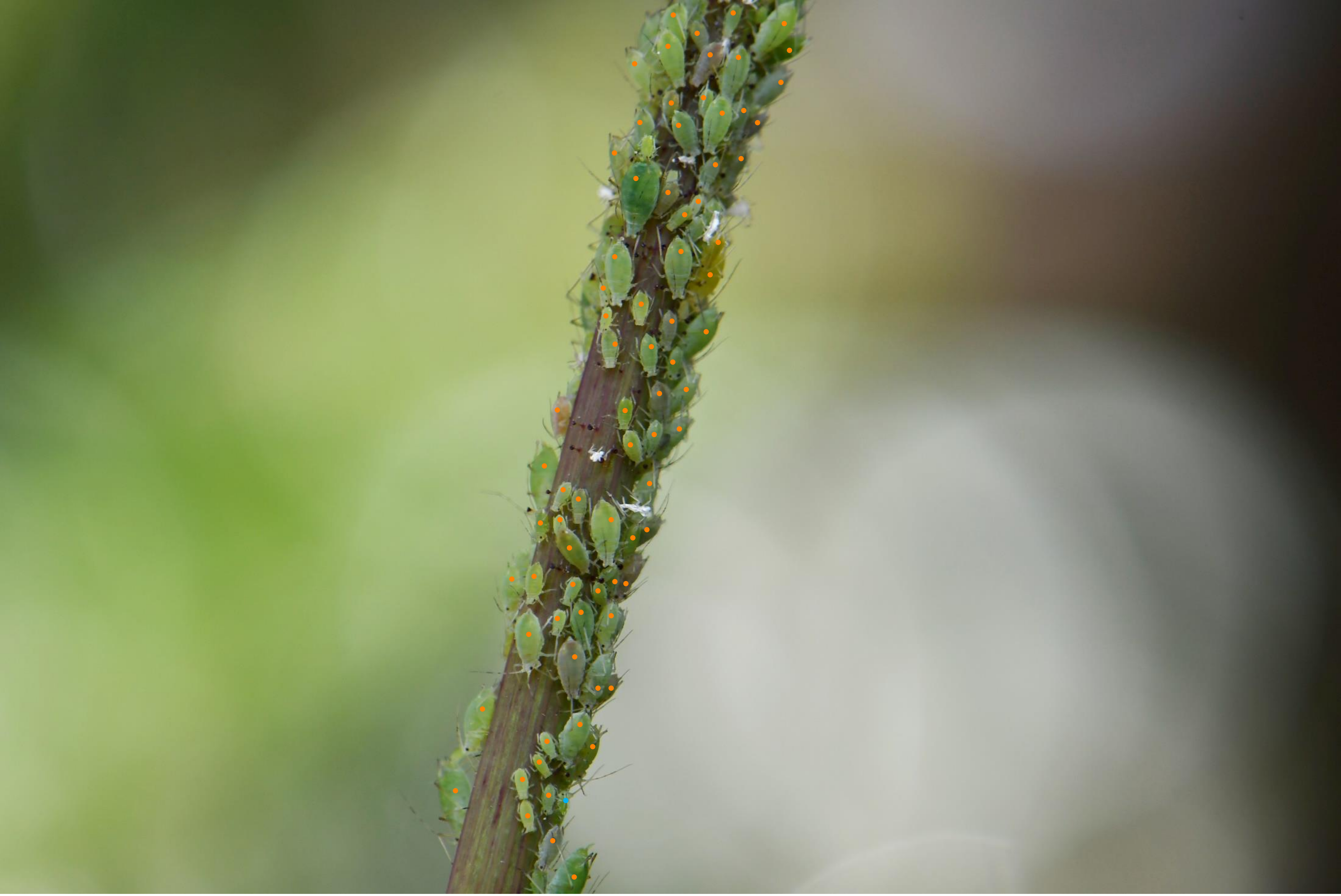

Picture ID: 8, *Hyperomyzus* sp. on *Sonchus* sp., 20 April 2022.

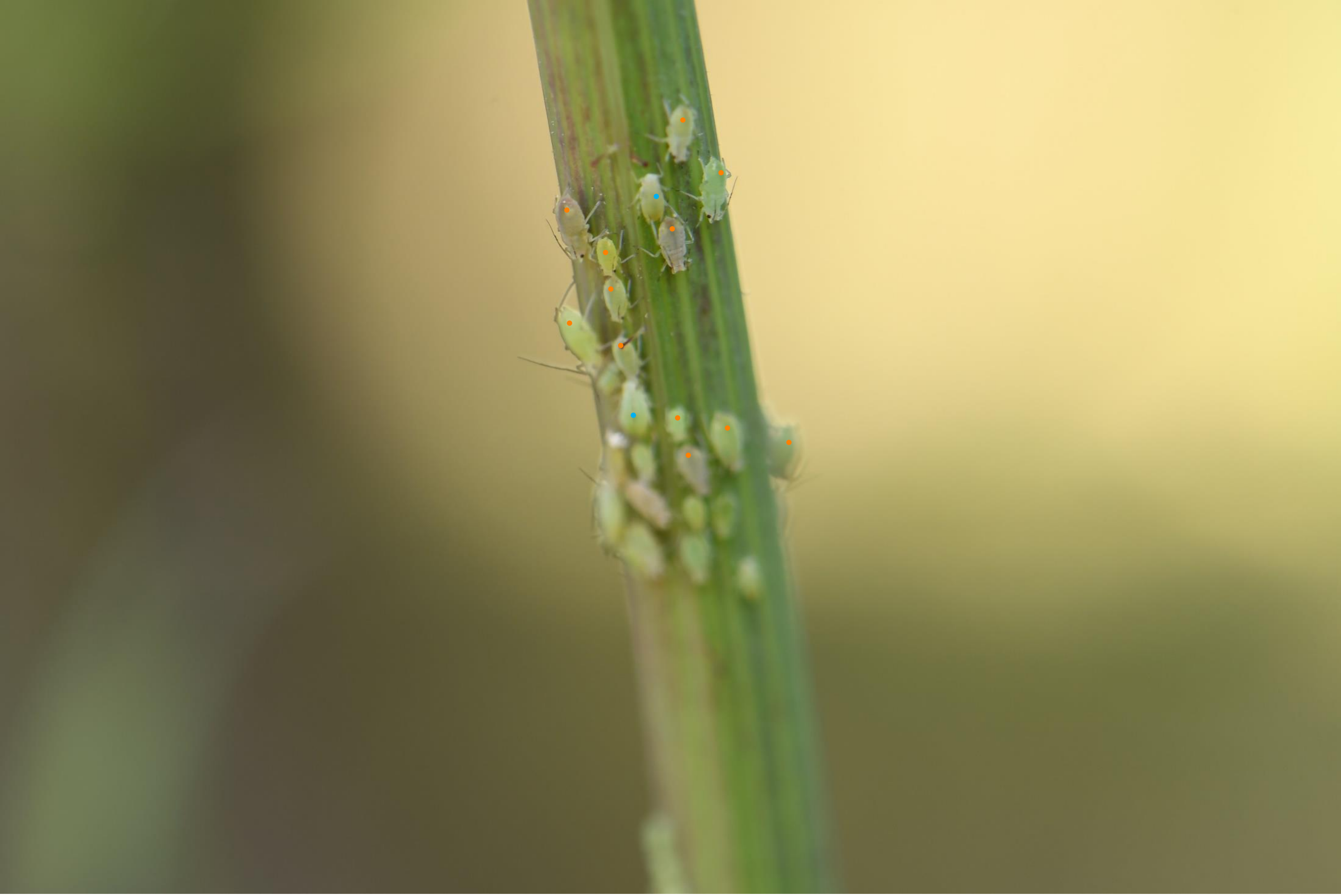

Picture ID: 9, *Hyperomyzus* sp. on *Sonchus* sp., 22 April 2022.

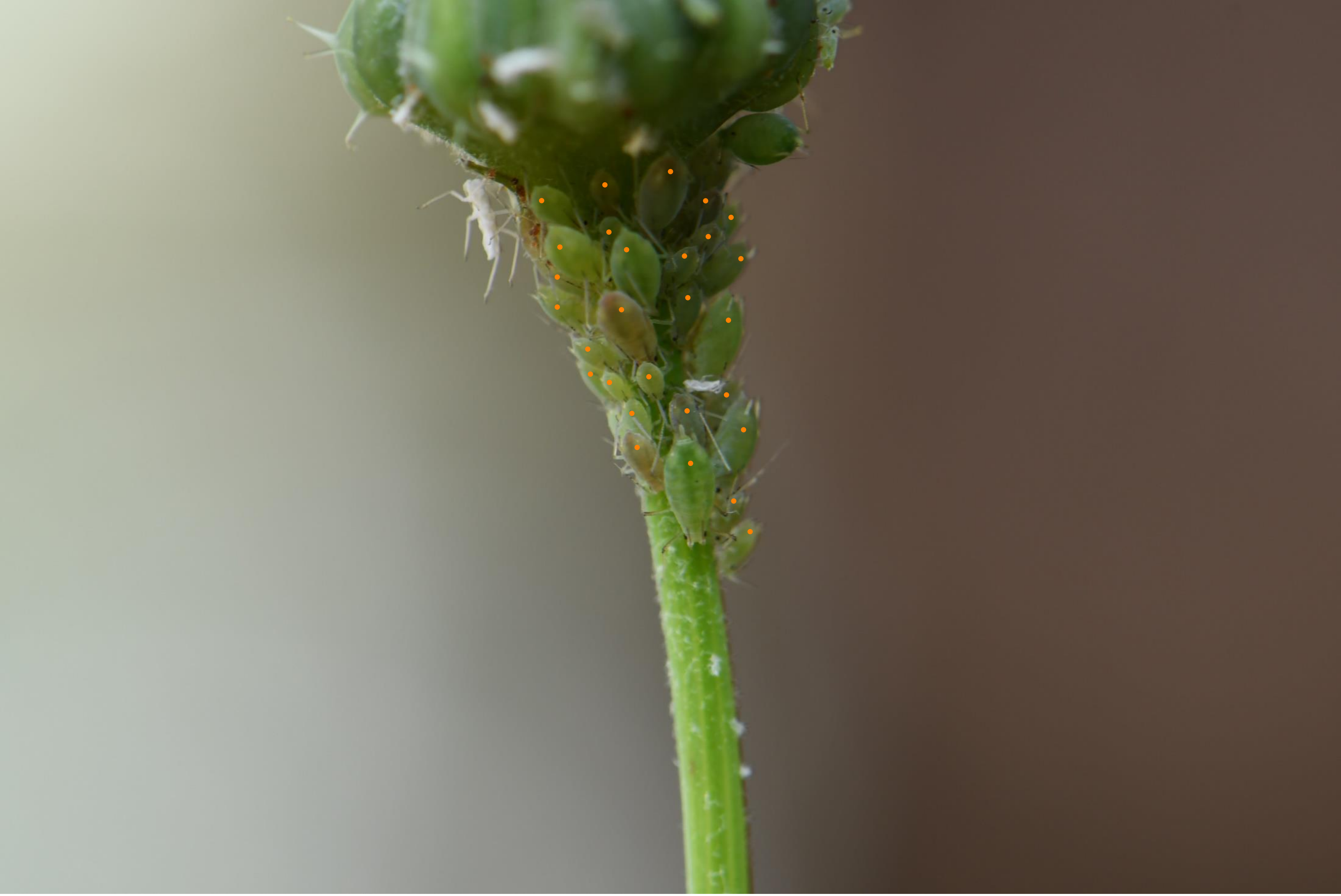

Picture ID: 10, *Hyperomyzus* sp. on *Sonchus* sp., 22 April 2022.

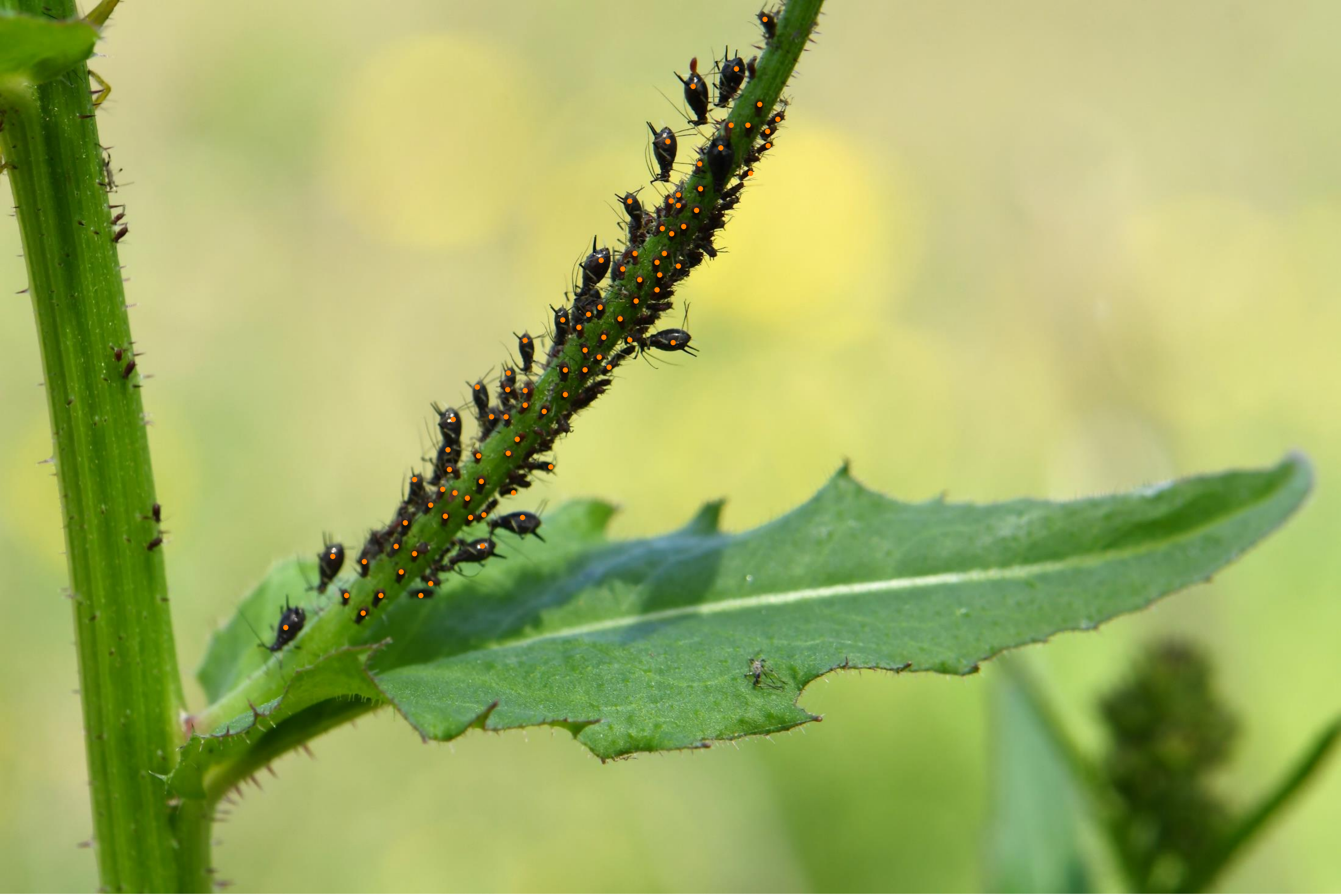

Picture ID: 11, *Uroleucon picridis* on *Picris hieracioides,* 22 April 2022.

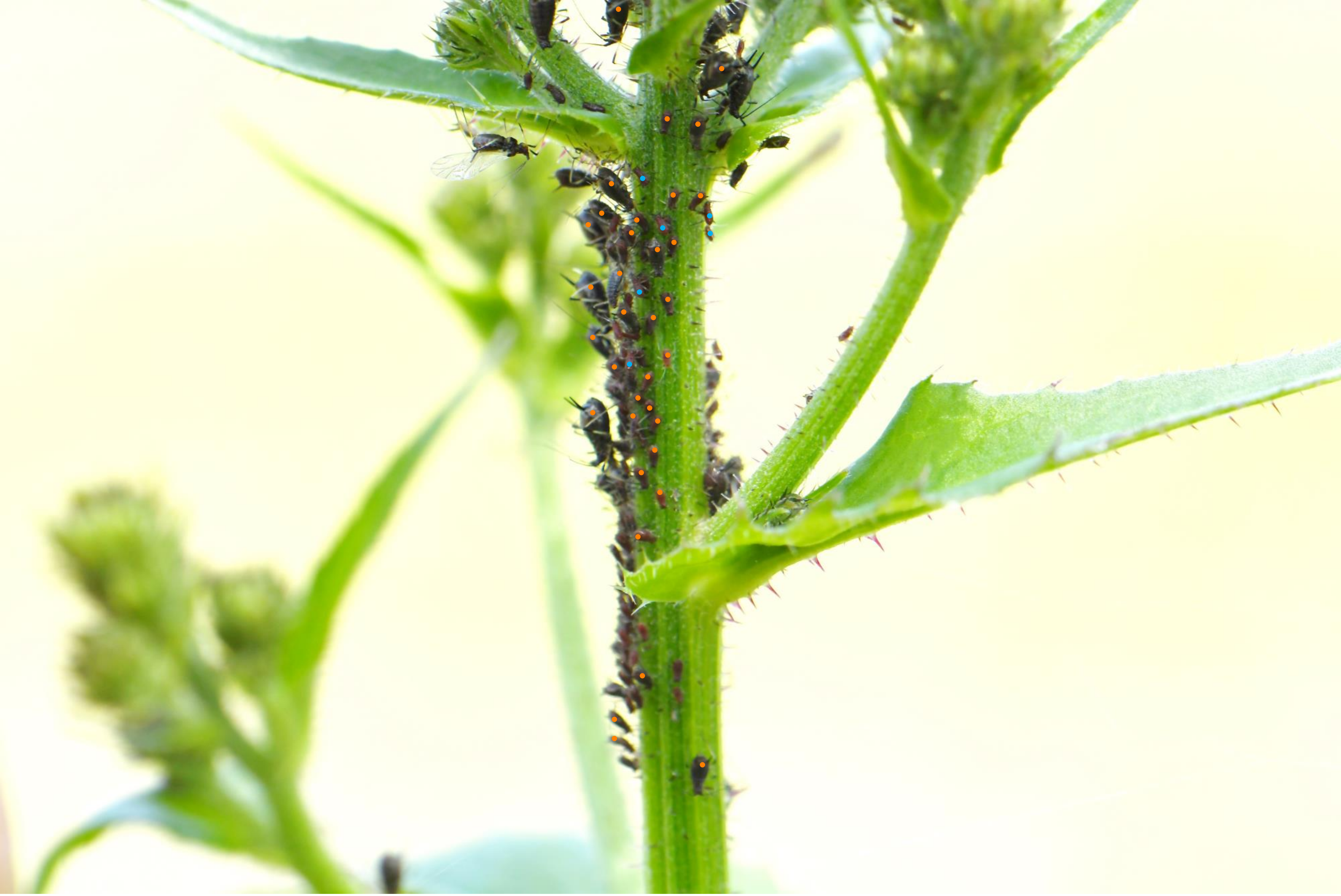

Picture ID: 12, *U. picridis* on *P. hieracioides,* 22 April 2022.

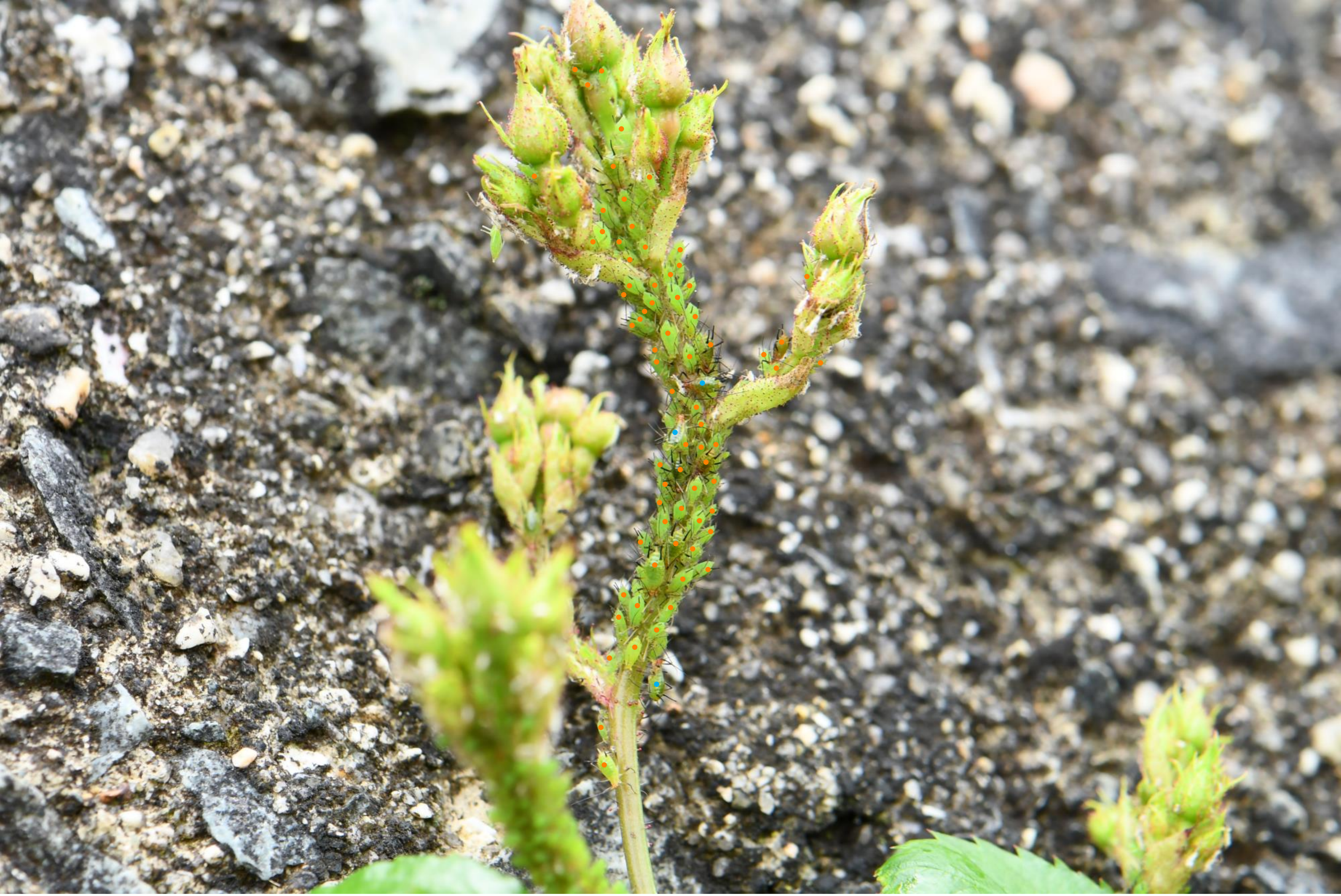

Picture ID: 13, *Sitobion ibarae* on *Rosa* sp., 24 April 2022.

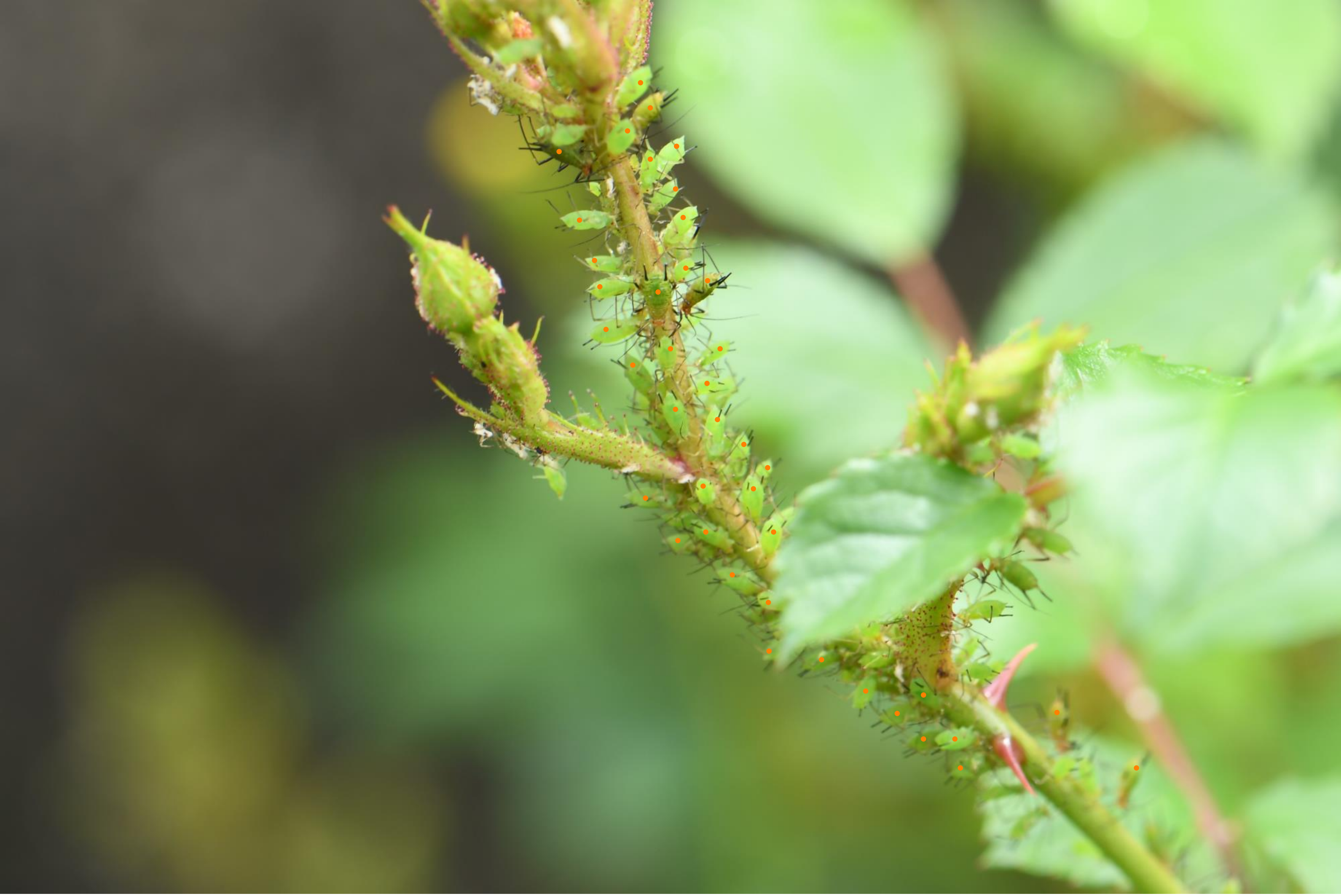

Picture ID: 14, *Si. ibarae* on *Rosa* sp., 24 April 2022.

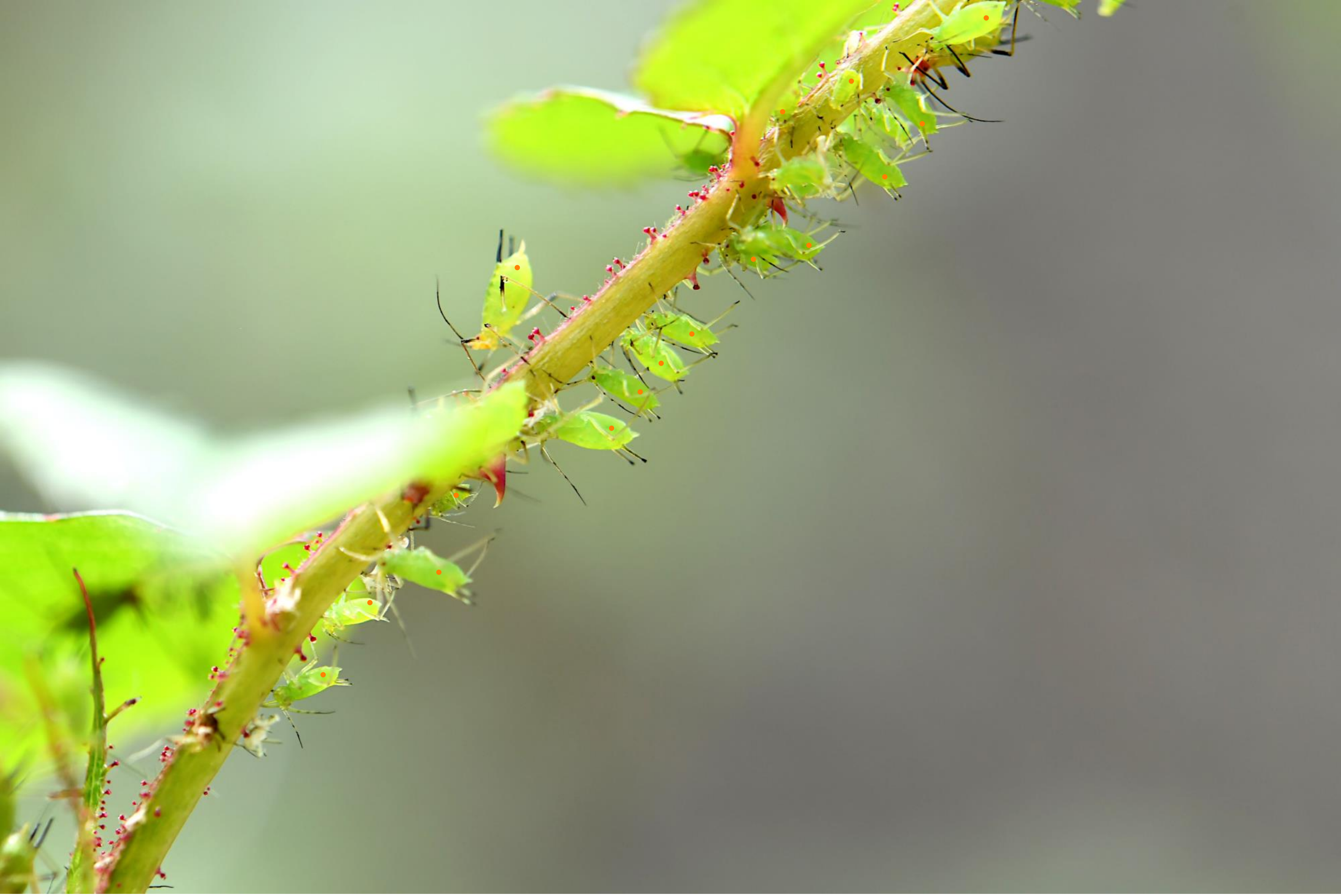

Picture ID: 15, *Si. ibarae* on *Rosa* sp., 24 April 2022.

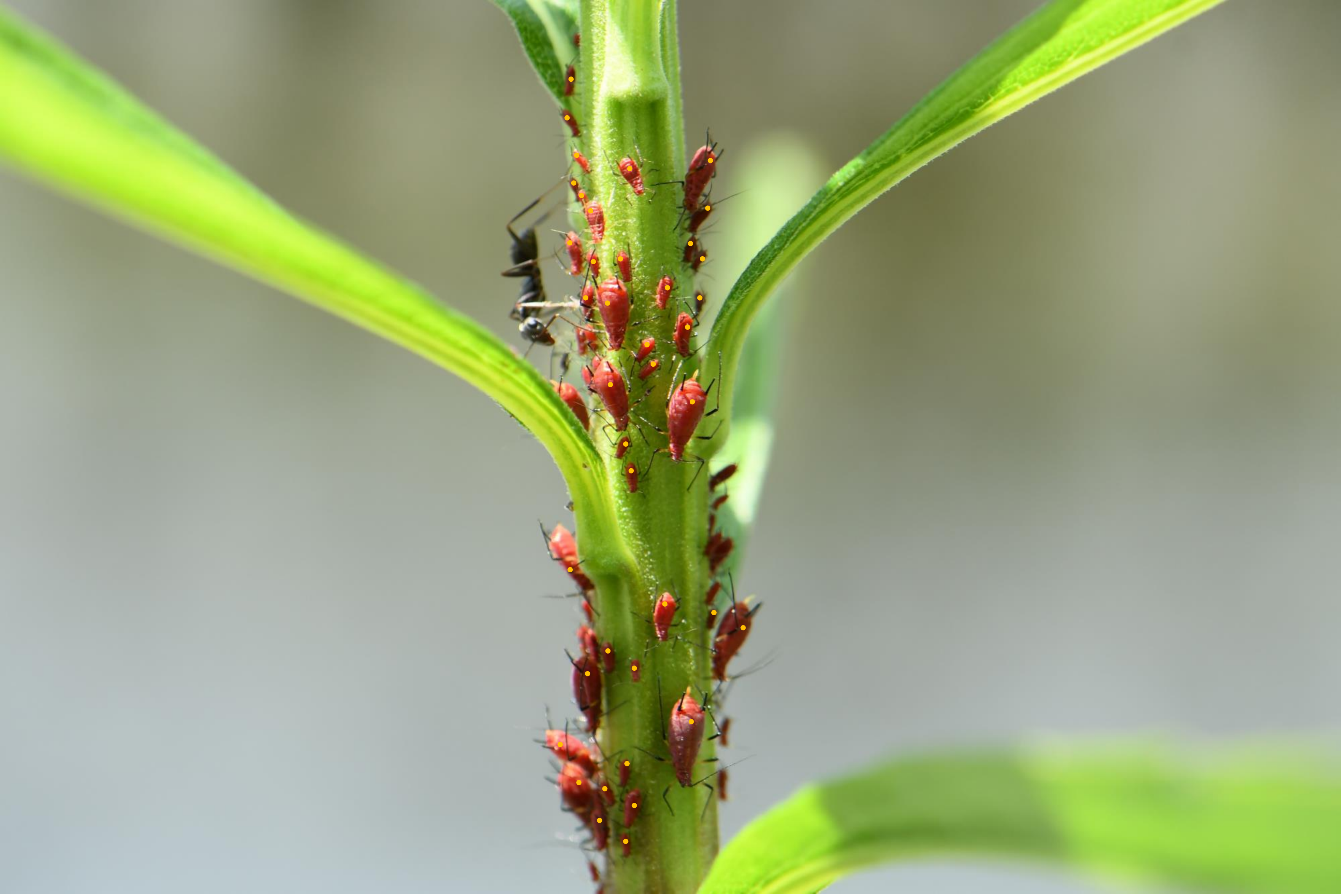

Picture ID: 16, *Uroleucon nigrotuberculatum* on *Solidago* sp., 8 May 2022.

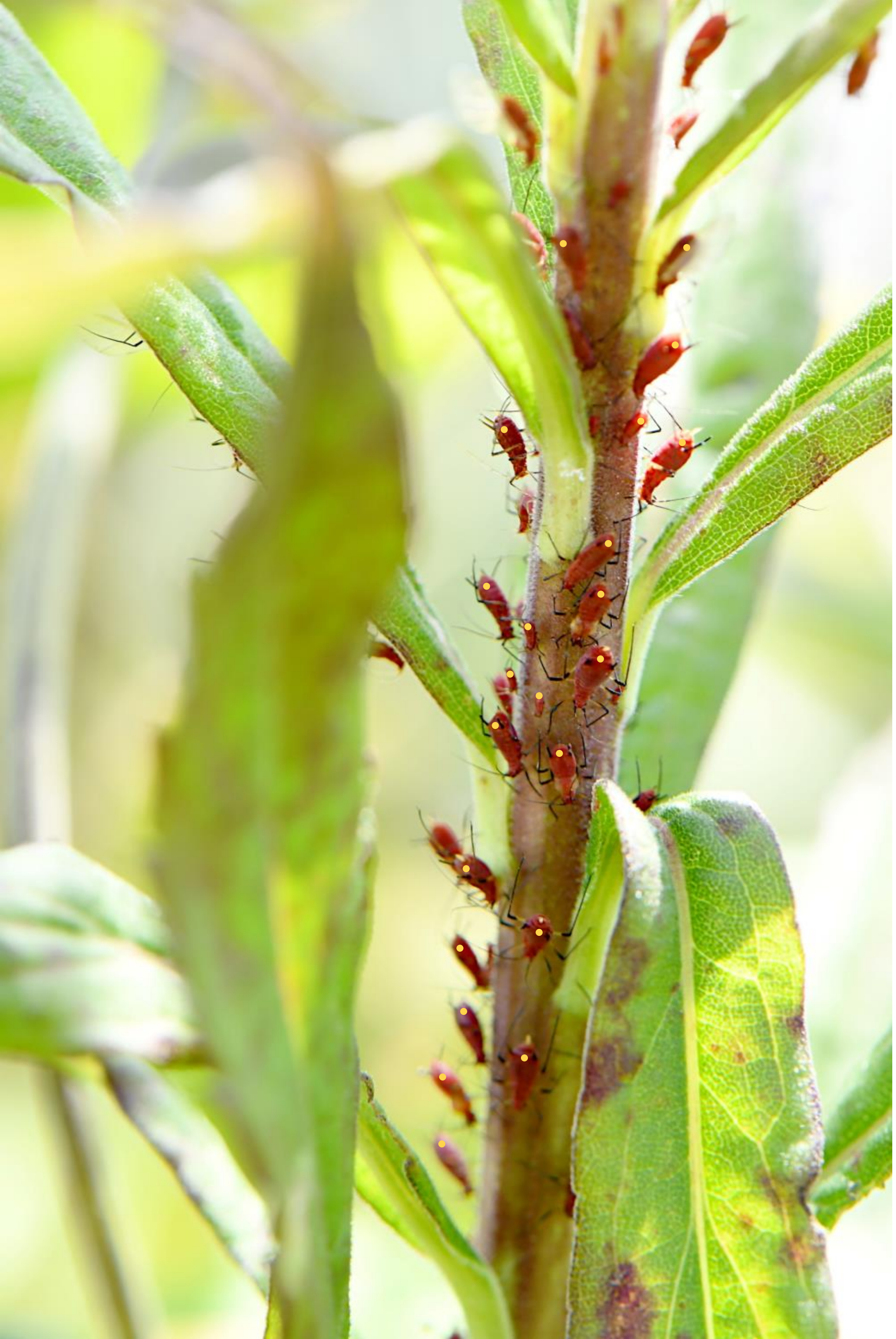

Picture ID: 17, *U. nigrotuberculatum* on *Solidago* sp., 8 May 2022.

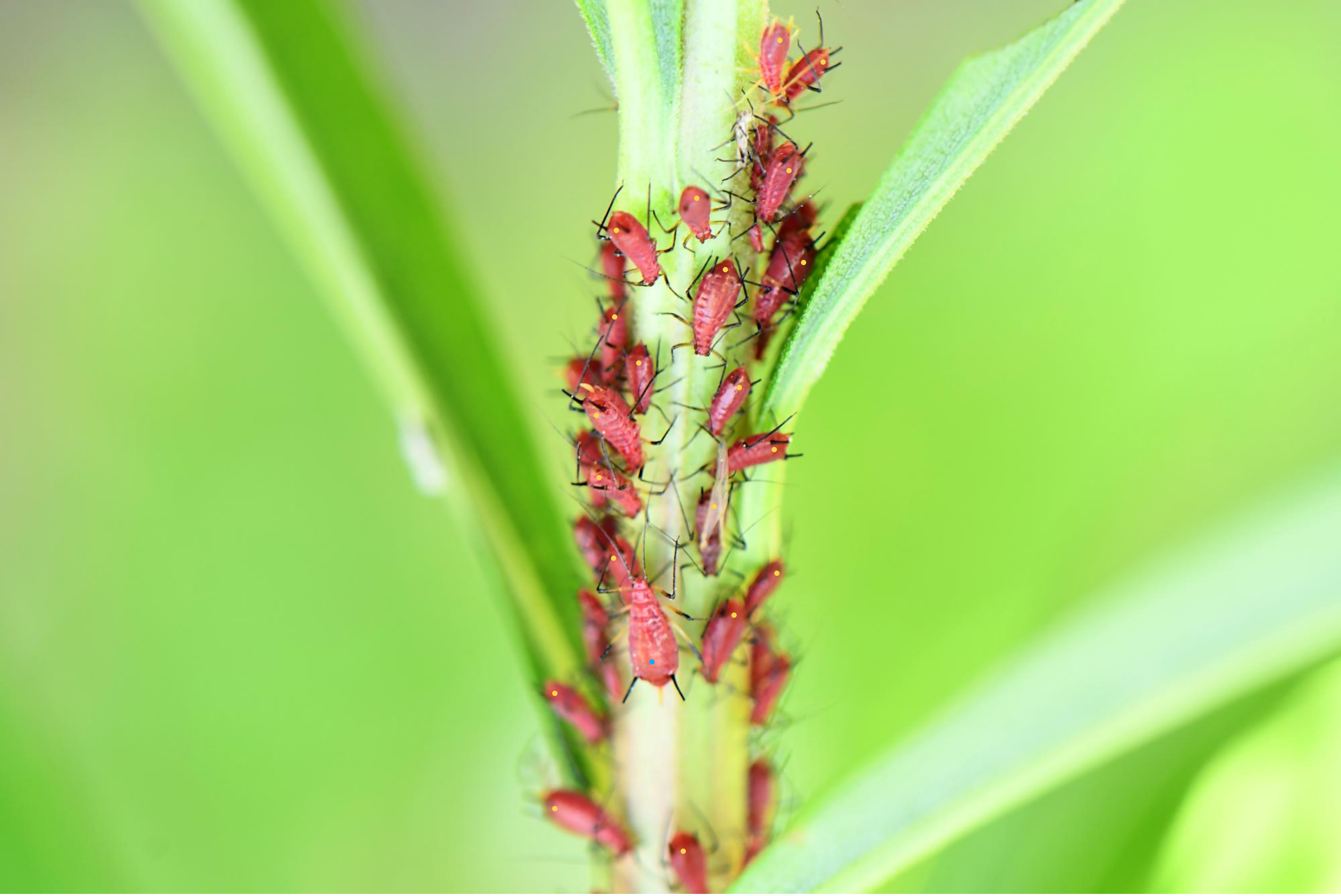

Picture ID: 18, *U. nigrotuberculatum* on *Solidago* sp., 8 May 2022.

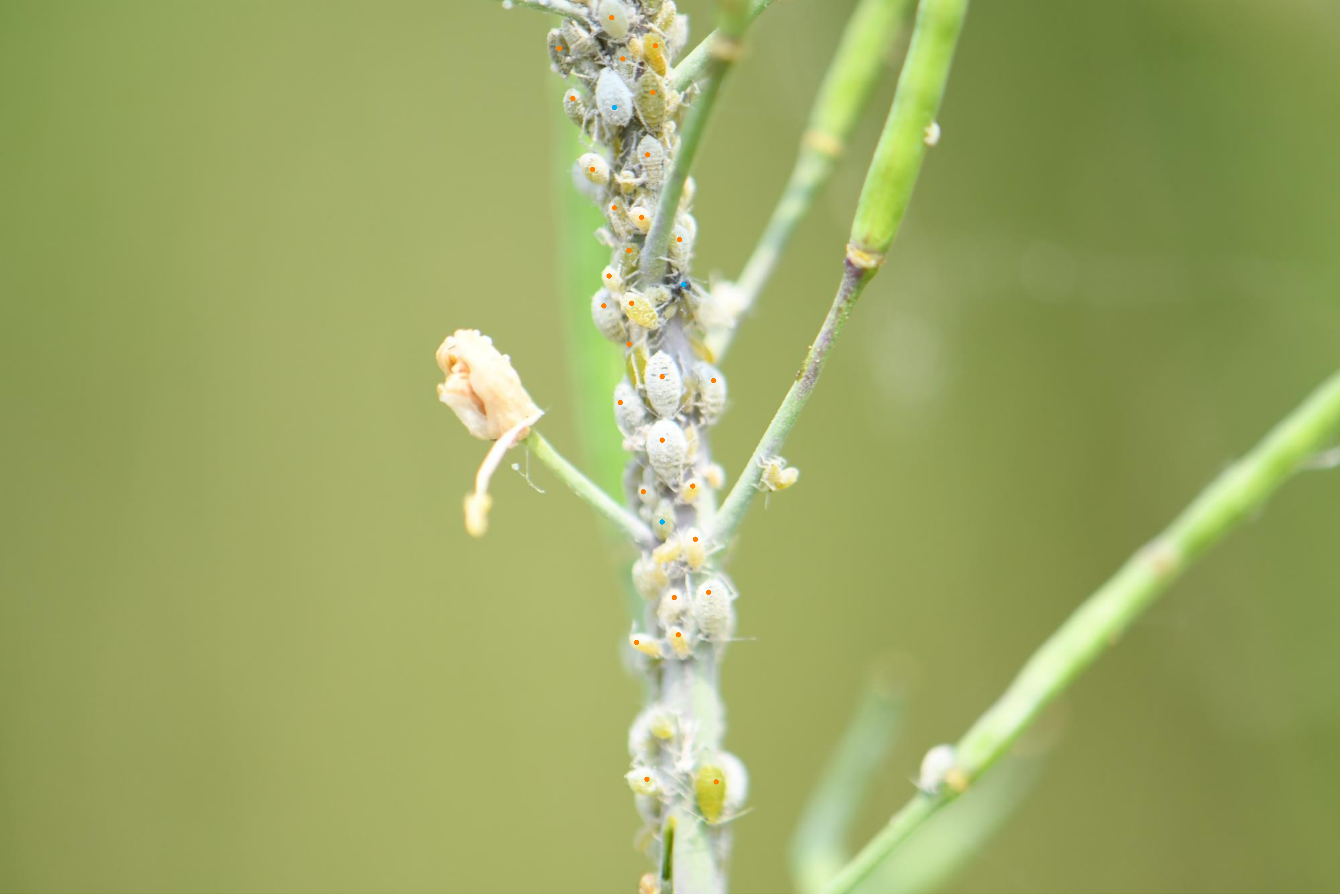

Picture ID: 19, *Brevicoryne brassicae* on *Brassica juncea*, 25 May 2022.

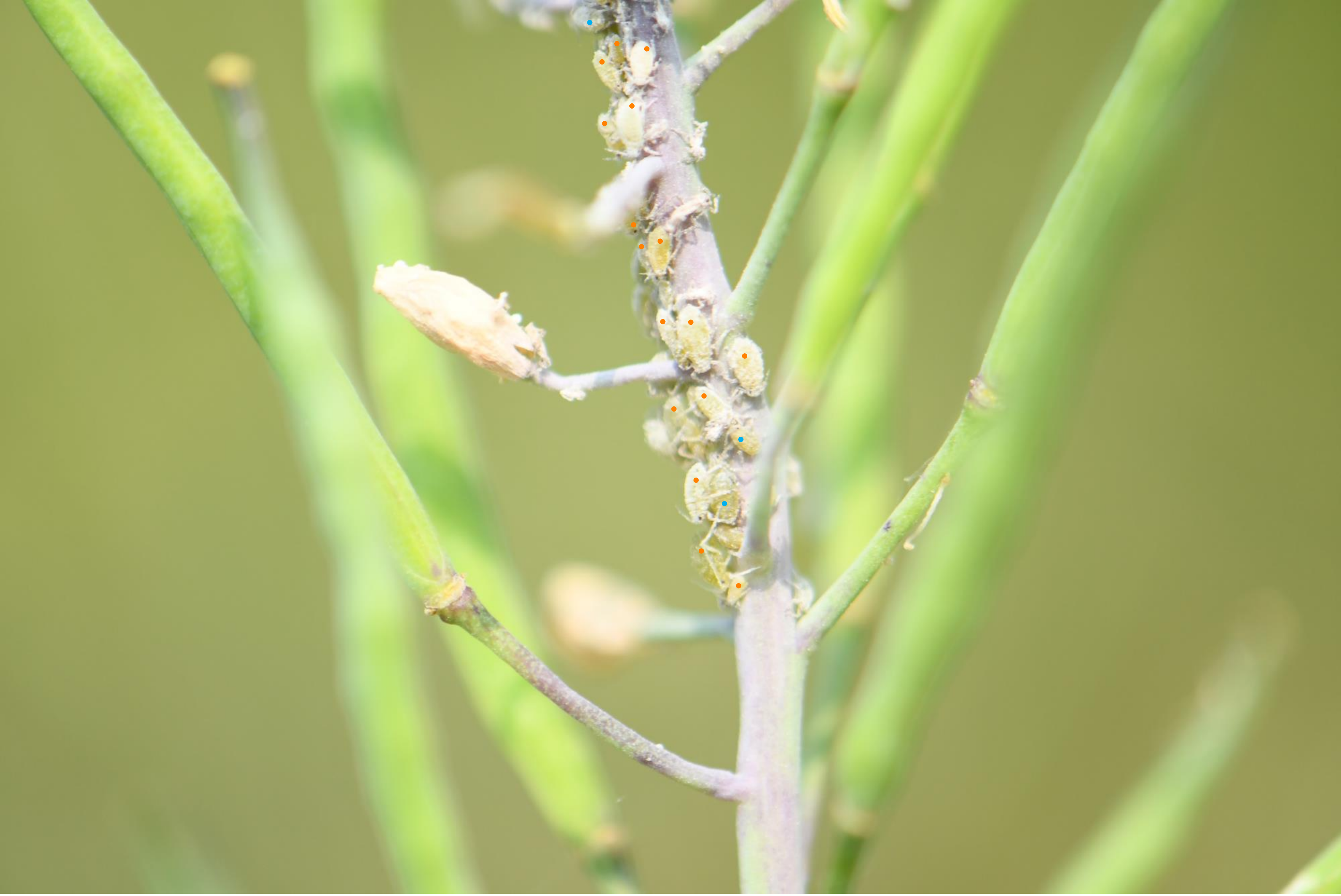

Picture ID: 20, *Bre. brassicae* on *Bra. juncea*, 25 May 2022.

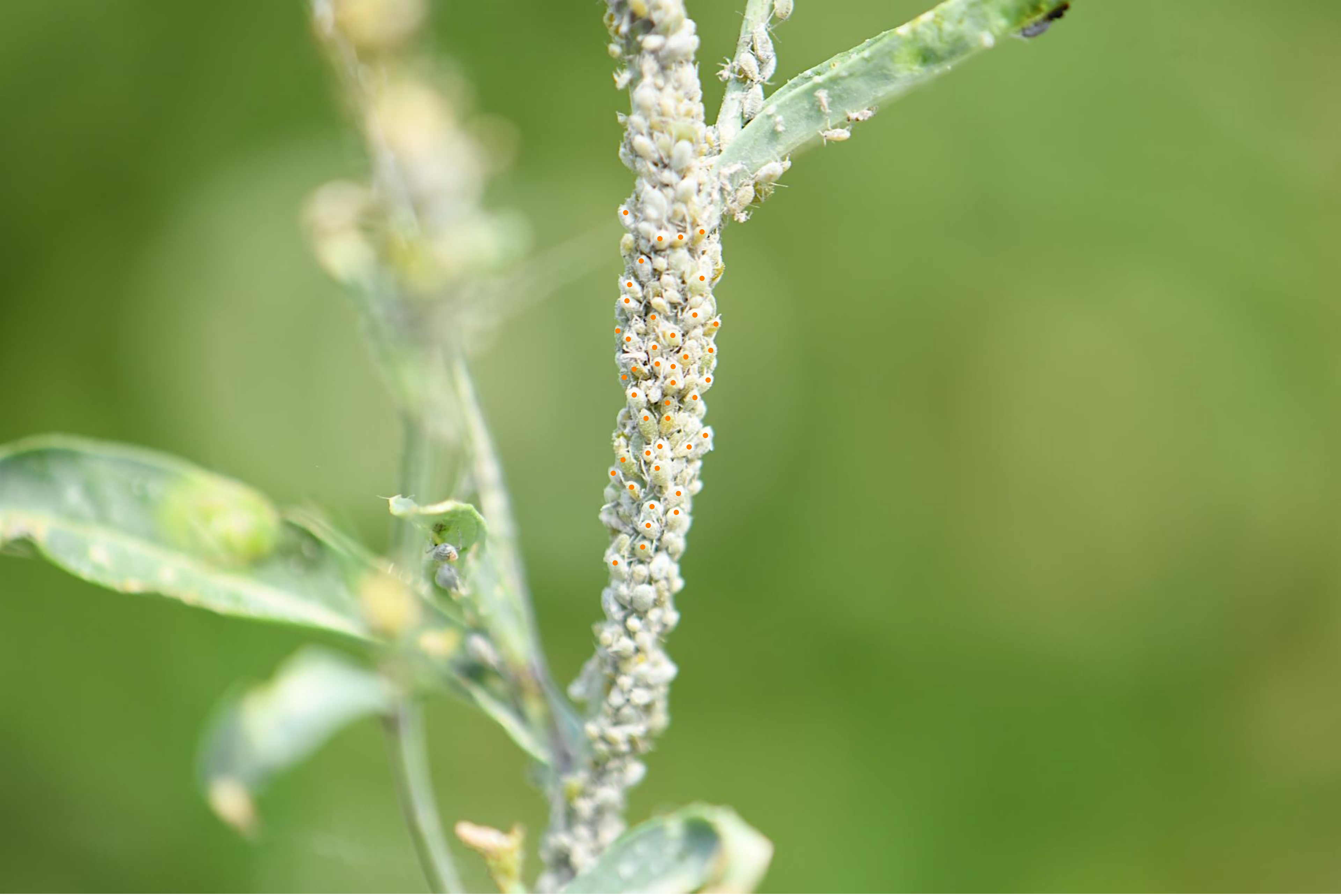

Picture ID: 21, *Bre. brassicae* on *Bra. juncea*, 25 May 2022.

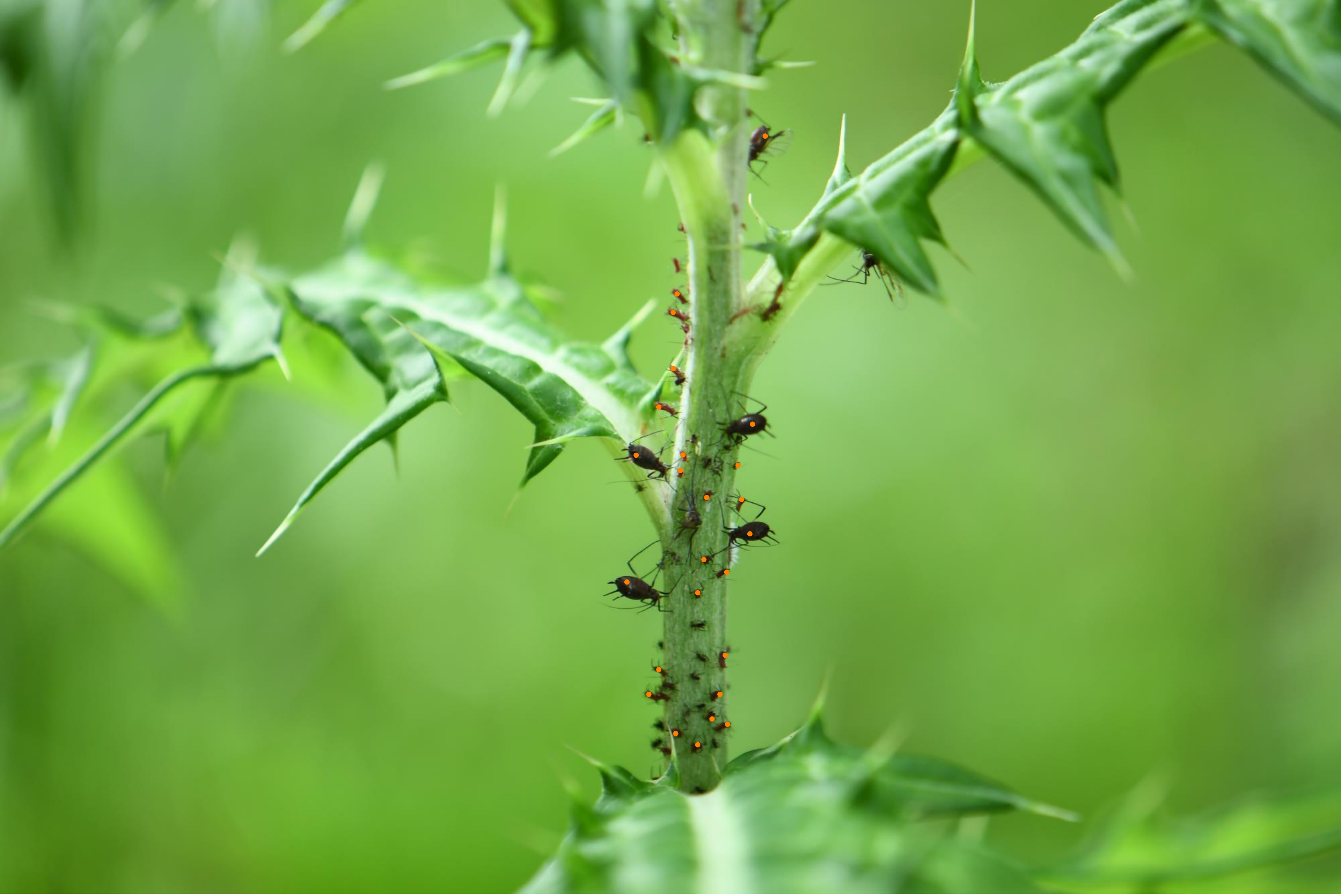

Picture ID: 22, *Uroleucon giganteum* on *Cirsium* sp., 25 June 2022.

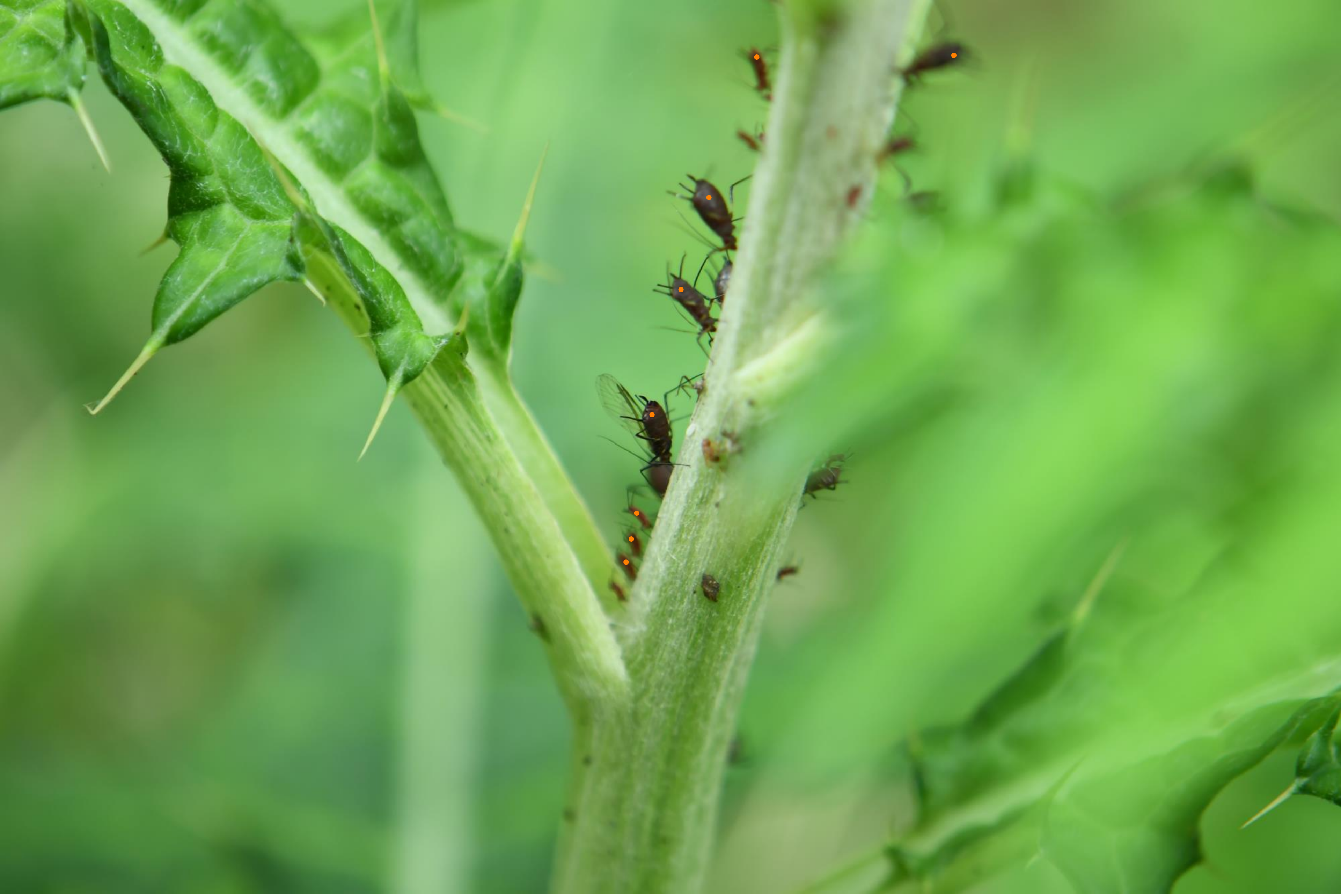

Picture ID: 23, *U. giganteum* on *Cirsium* sp., 25 June 2022.

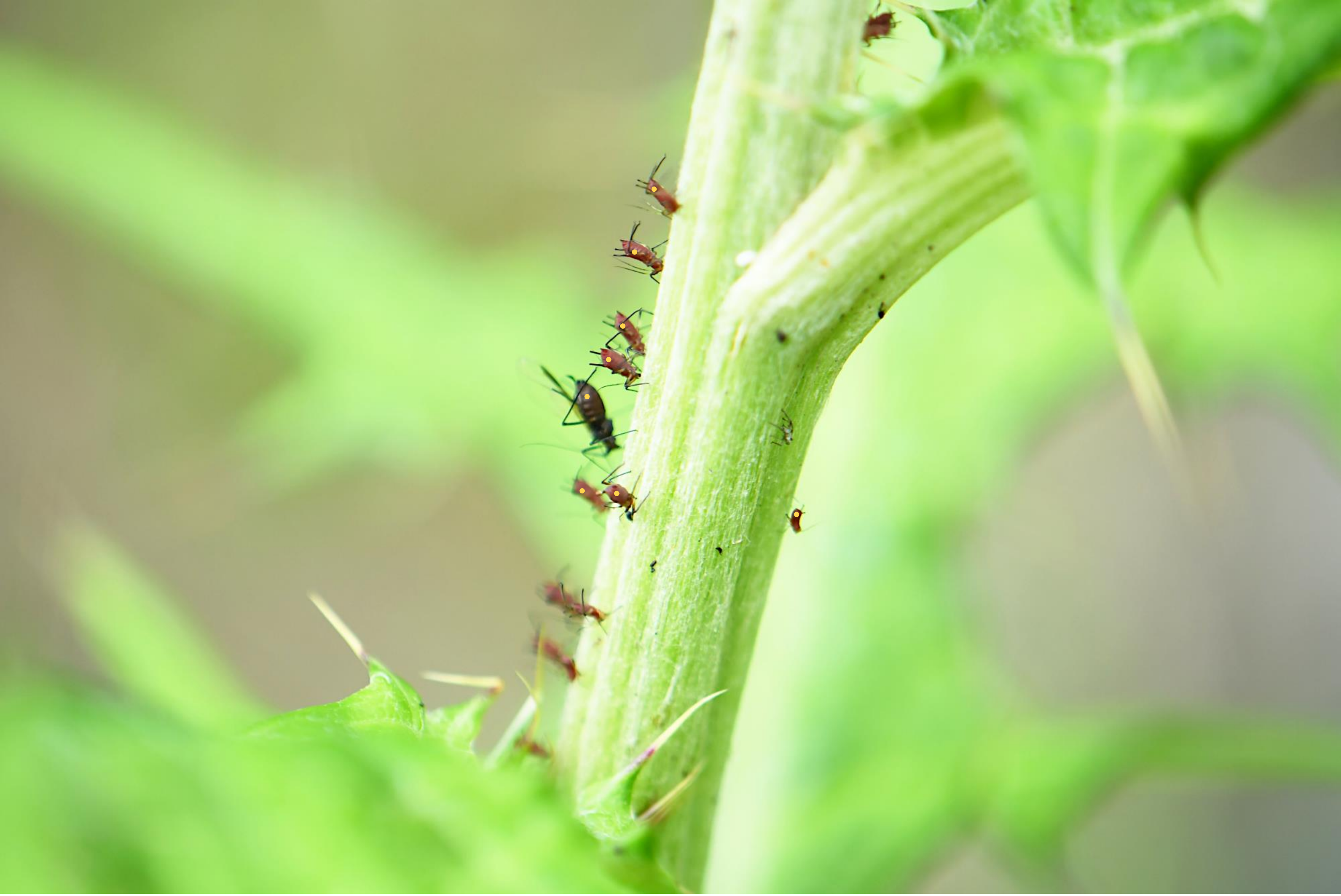

Picture ID: 24, *U. giganteum* on *Cirsium* sp., 25 June 2022.

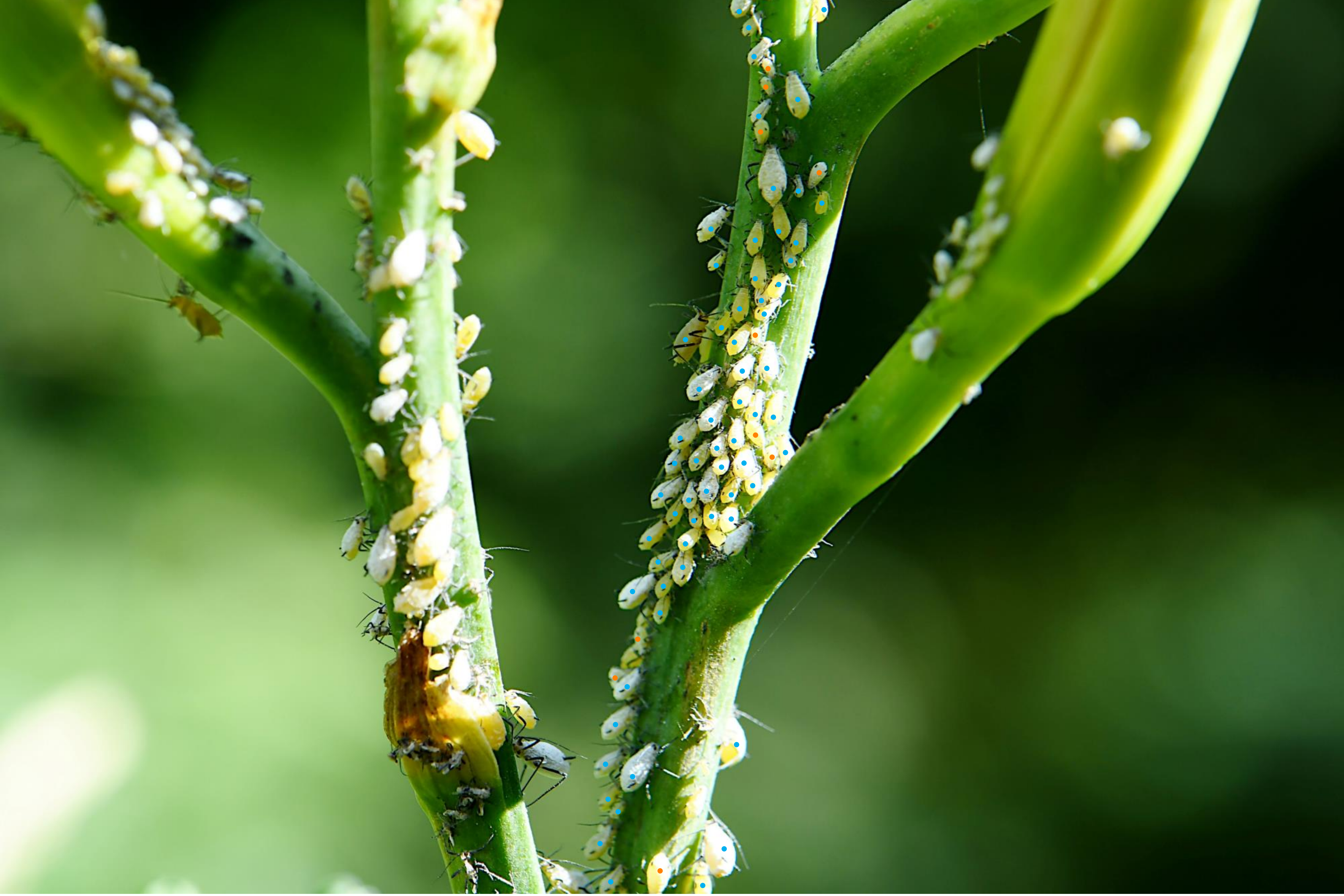

Picture ID: 25, *Indomegoura indica* on *Hemerocallis* sp., 2 July 2023.

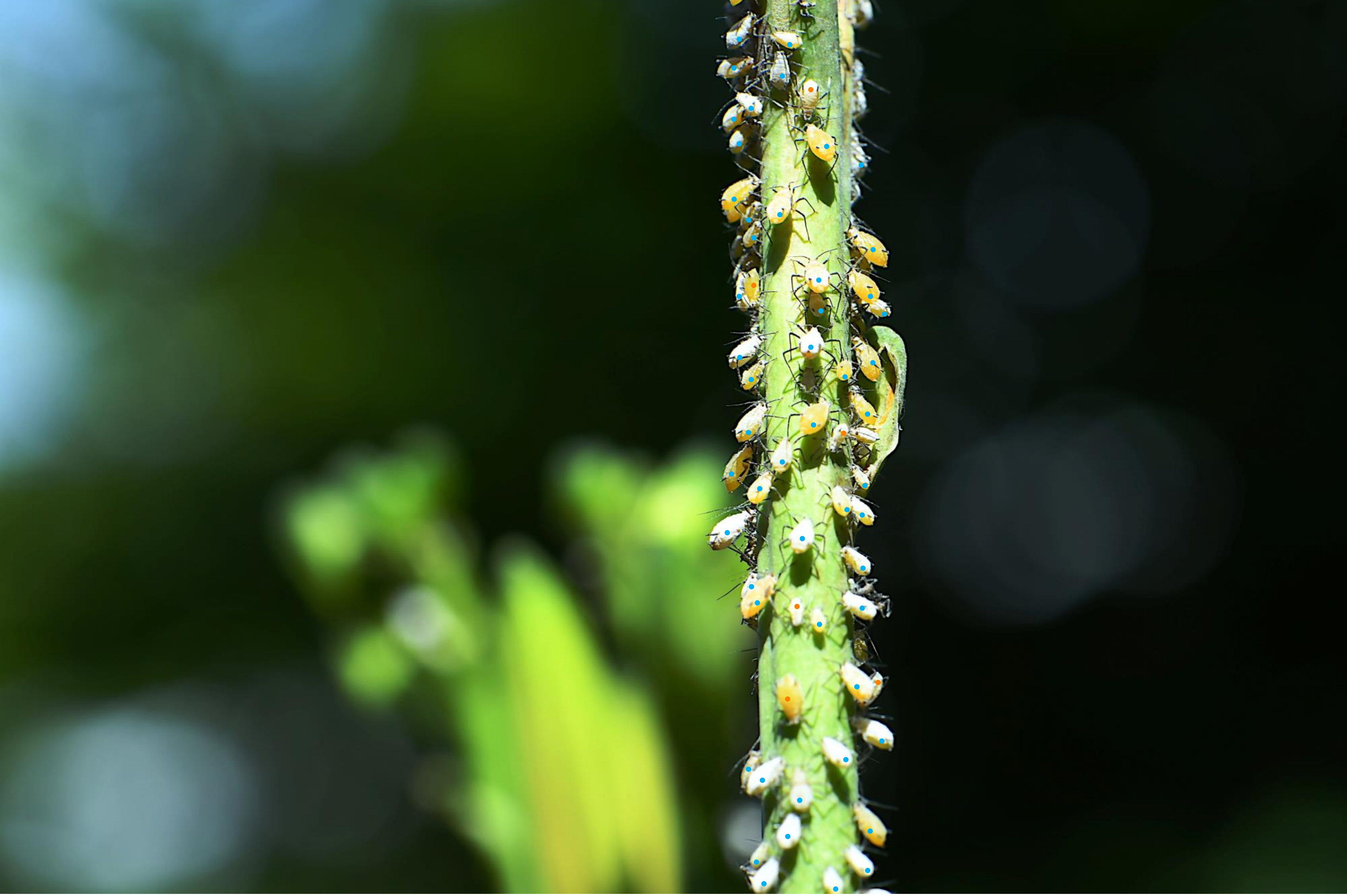

Picture ID: 26, *I. indica* on *Hemerocallis* sp., 2 July 2023.

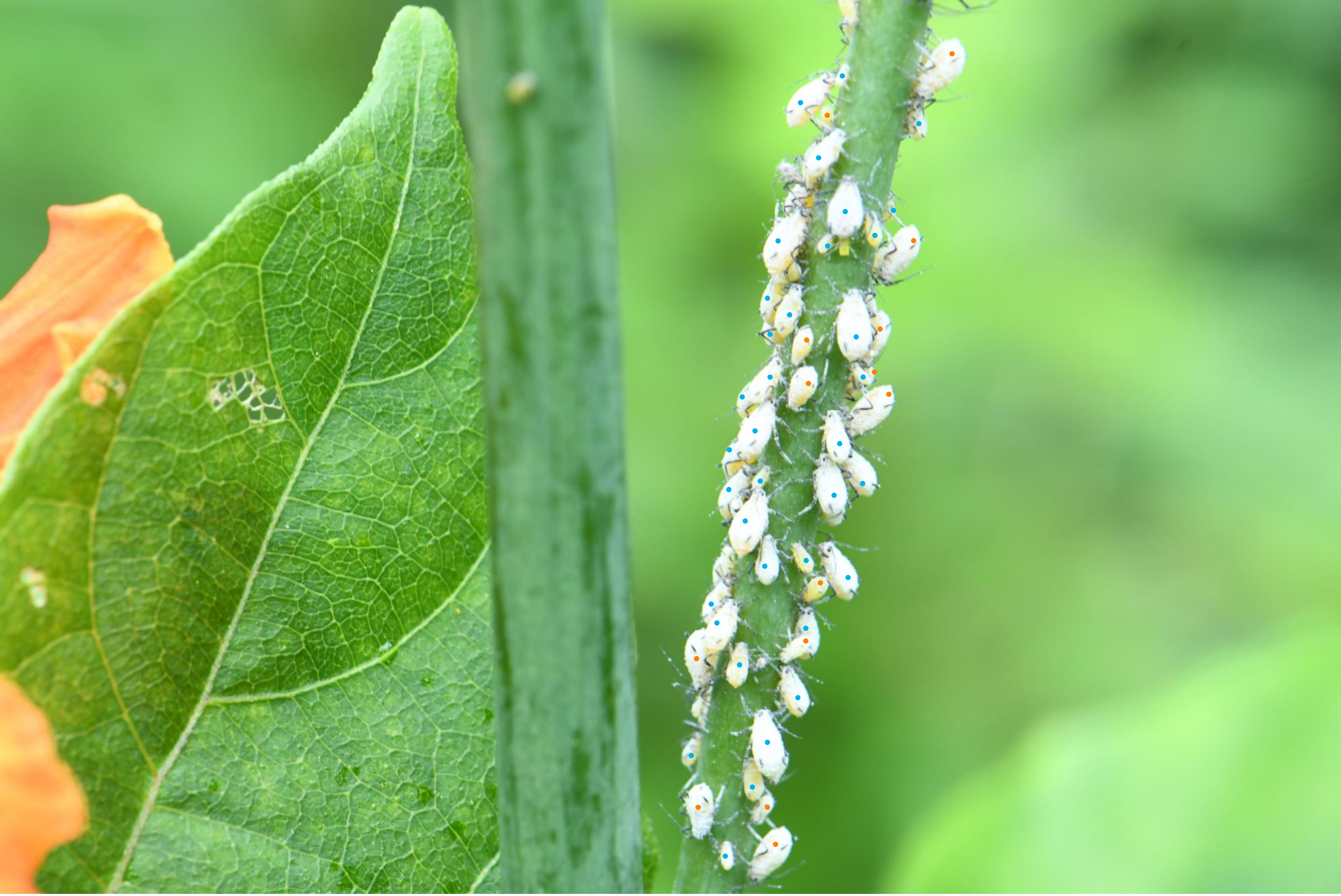

Picture ID: 27, *I. indica* on *Hemerocallis* sp., 16 July 2023.

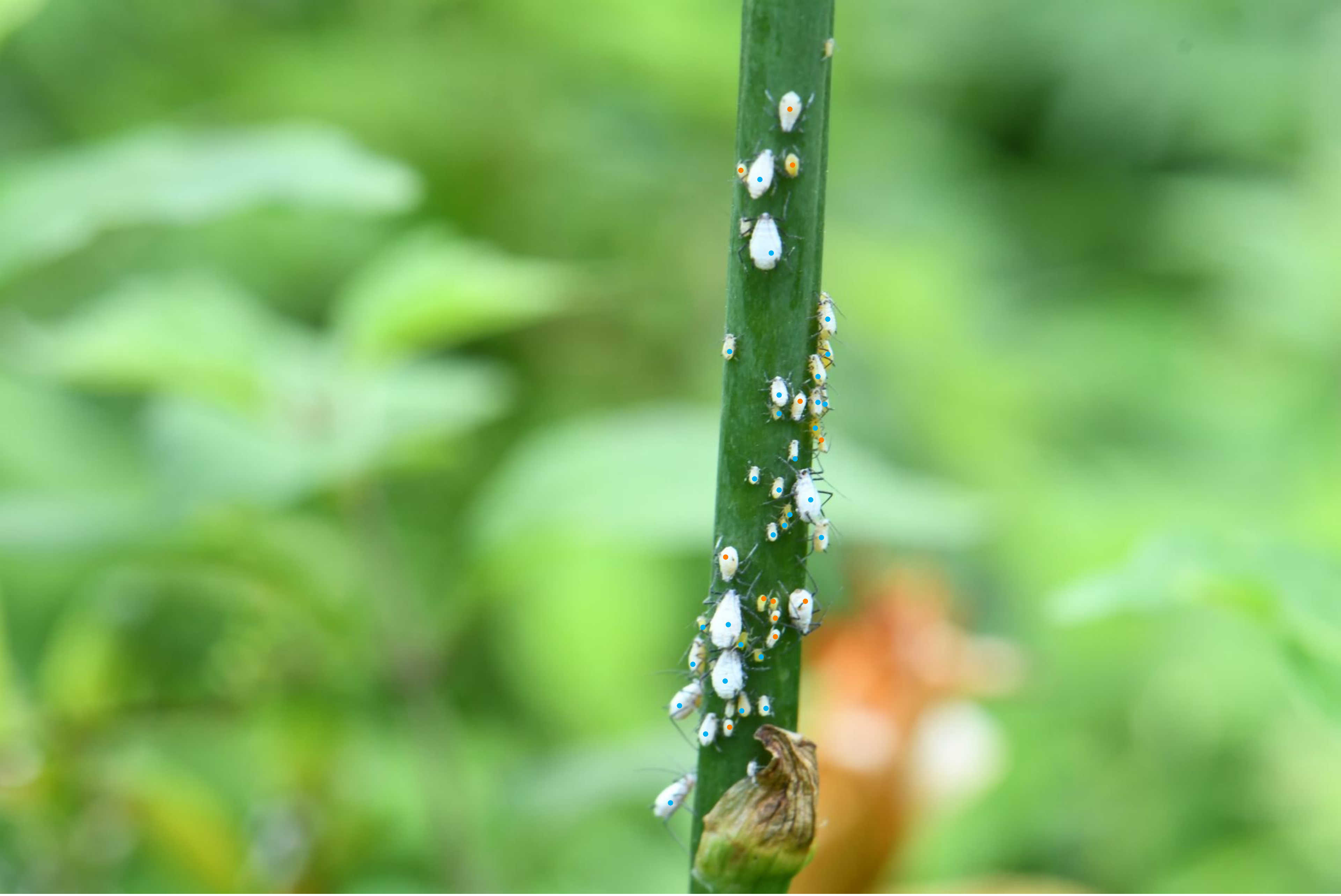

Picture ID: 28, *I. indica* on *Hemerocallis* sp., 16 July 2023.

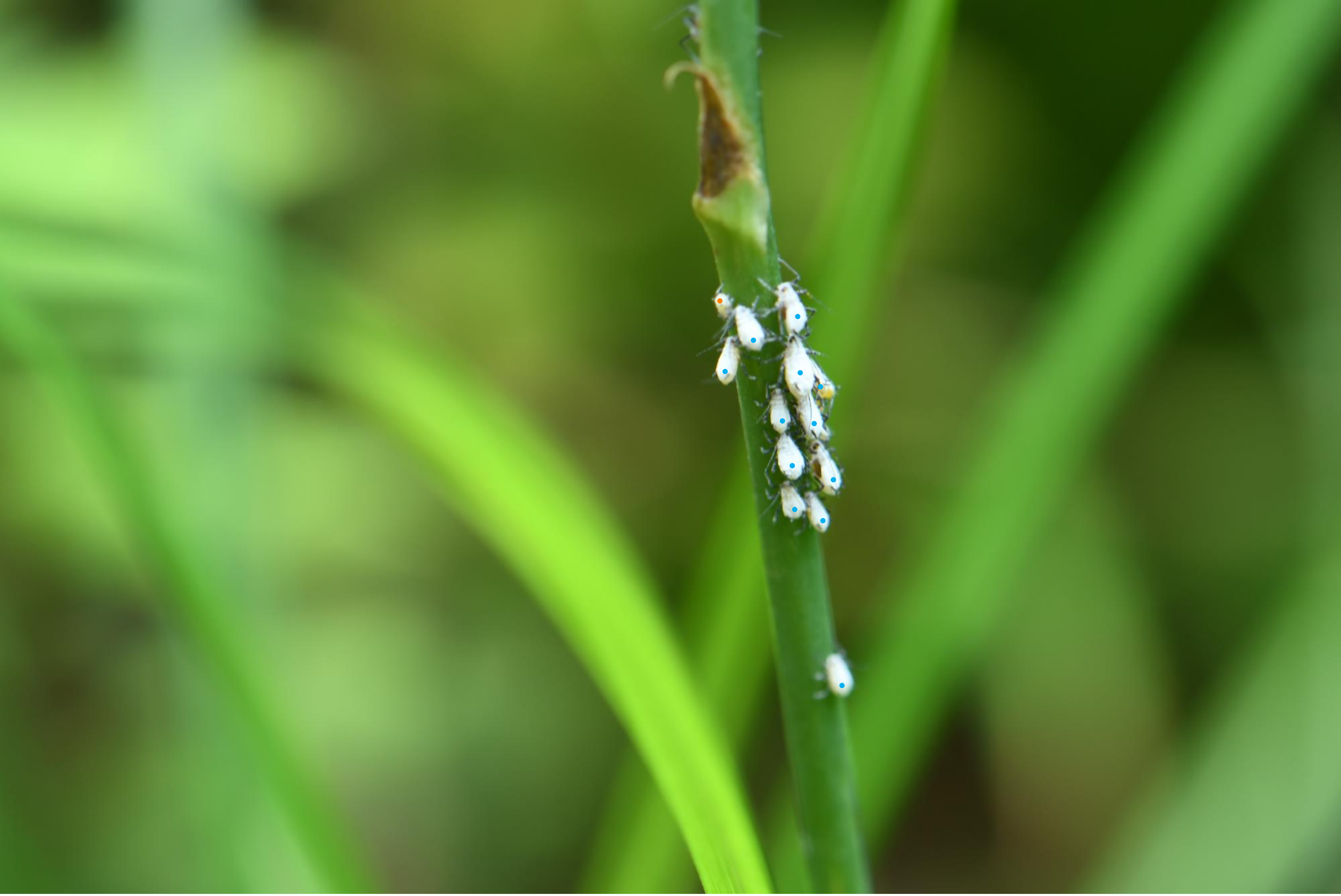

Picture ID: 29, *I. indica* on *Hemerocallis* sp., 16 July 2023.

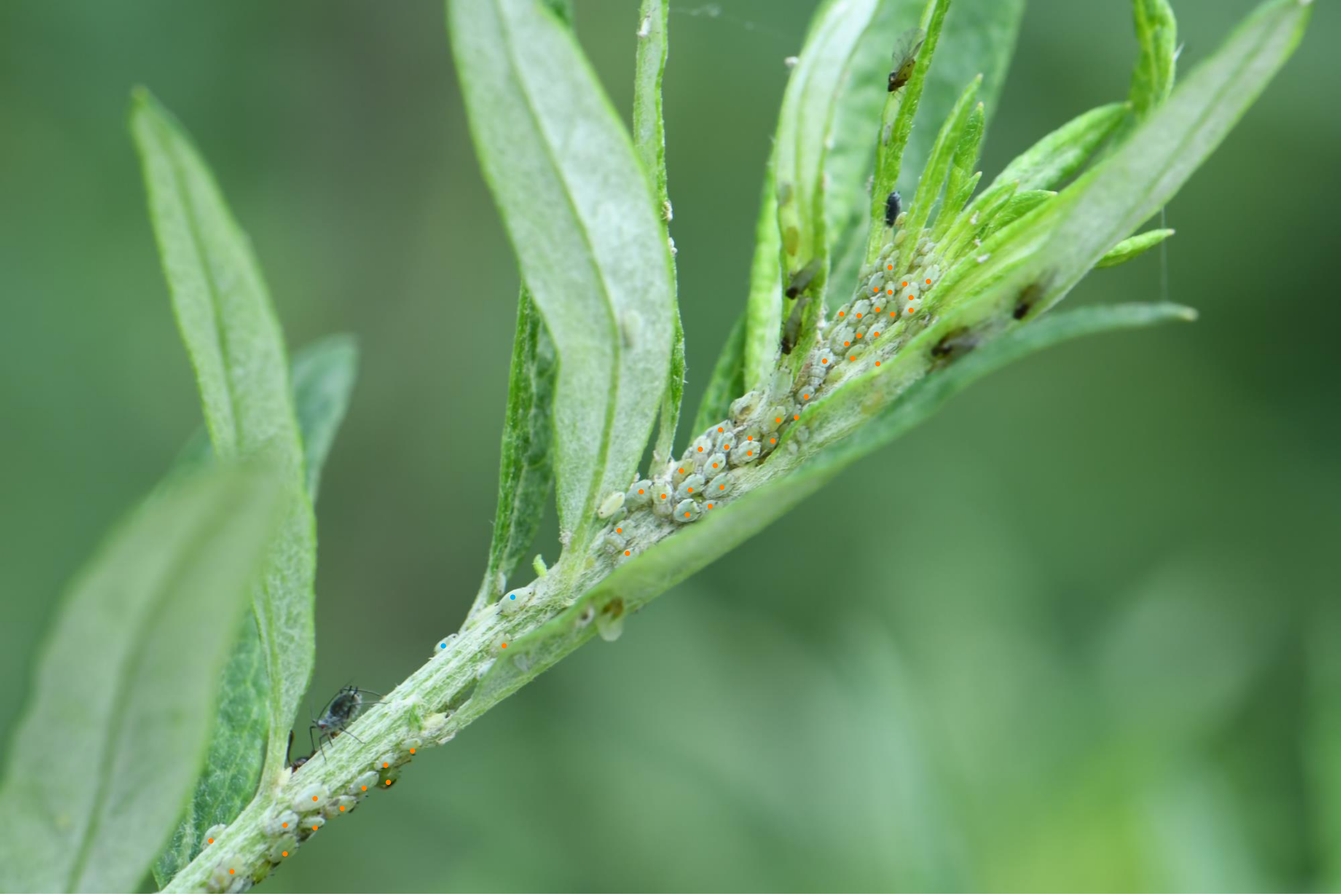

Picture ID: 30, *Aphis kurosawai* on *Artemisia indica*, 16 July 2023.

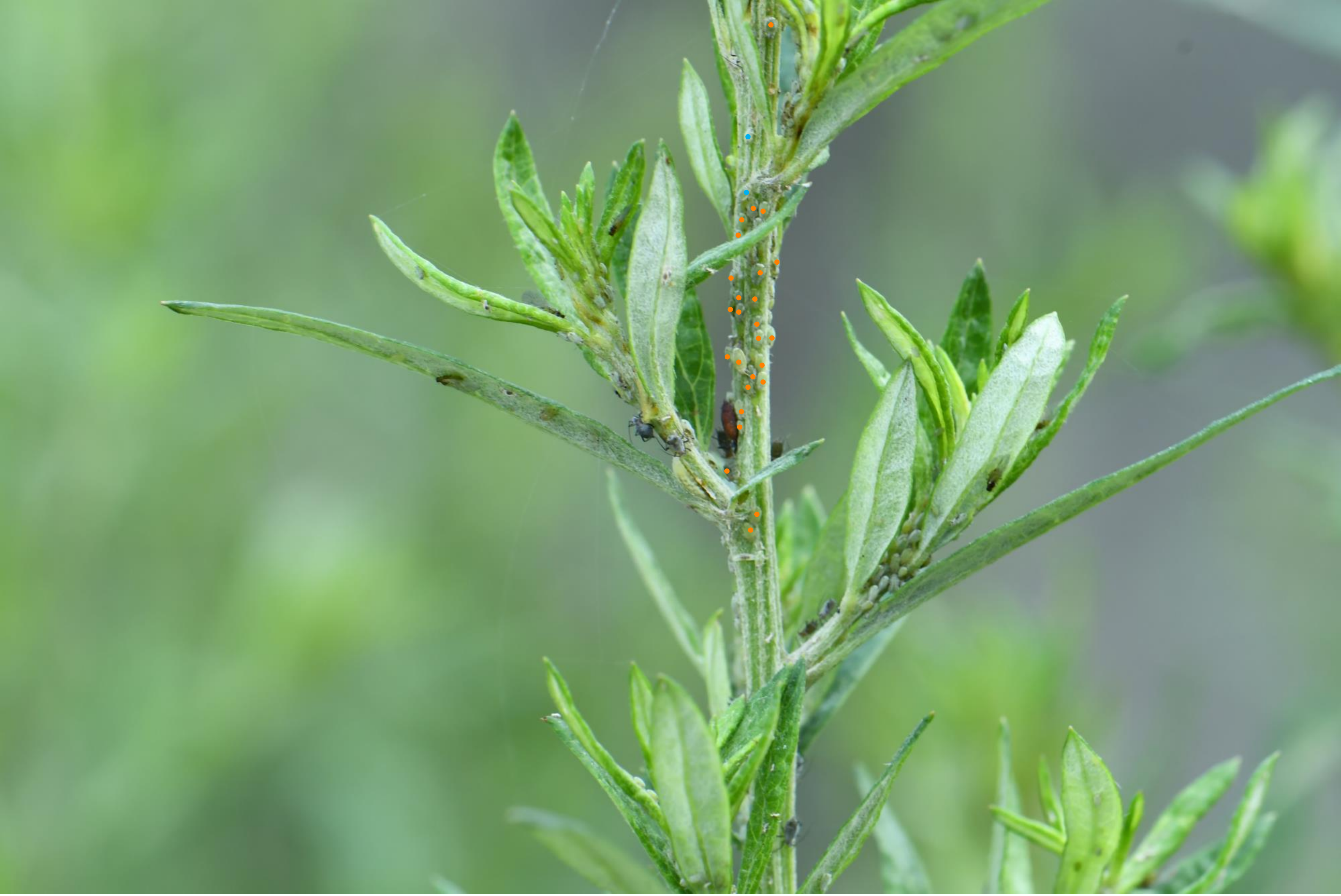

Picture ID: 31, *Ap. kurosawai* on *Ar. indica*, 16 July 2023.

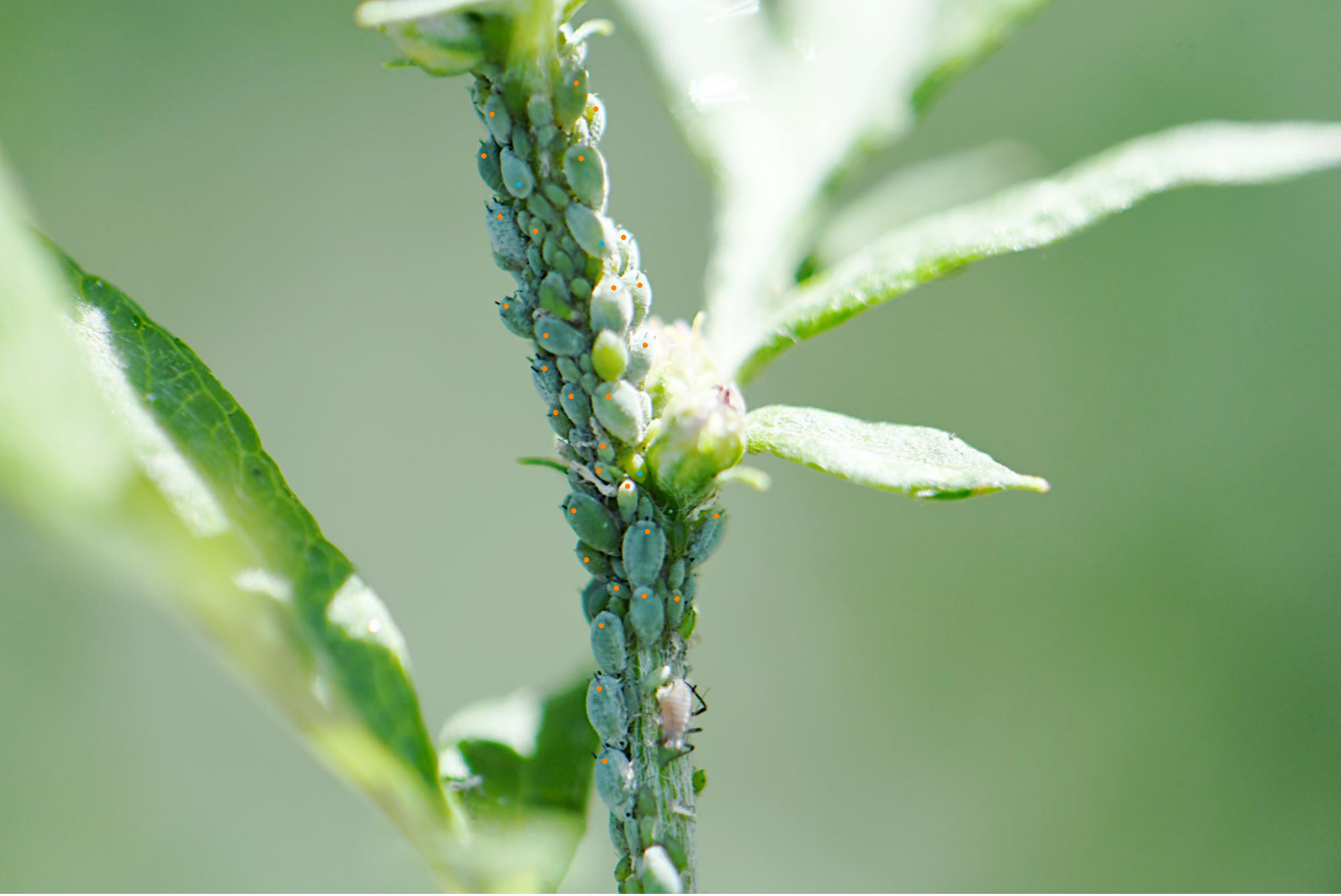

Picture ID: 32, *Ap. kurosawai* on *Ar. indica*, 20 October 2024.

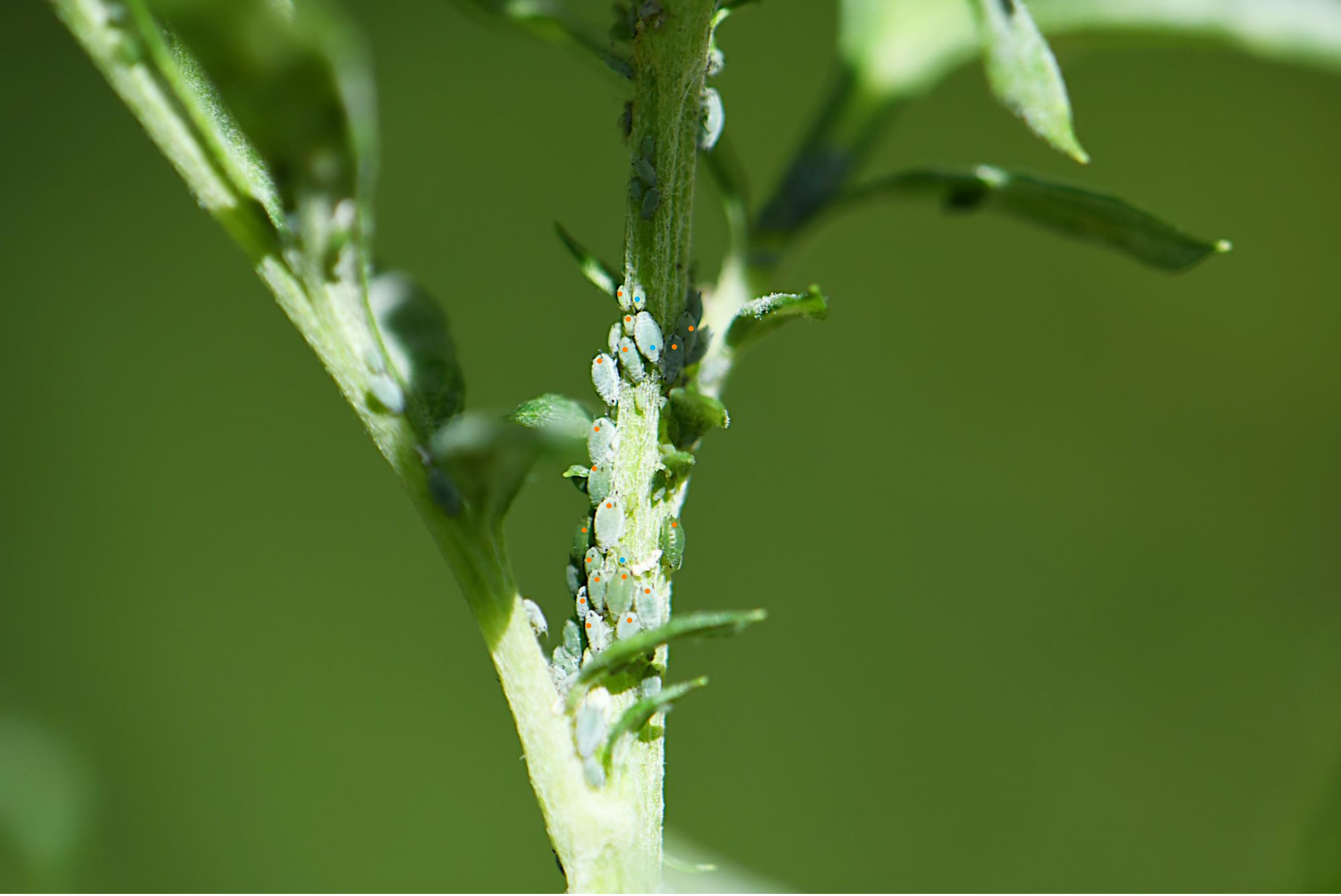

Picture ID: 33, *Ap. kurosawai* on *Ar. indica*, 20 October 2024.

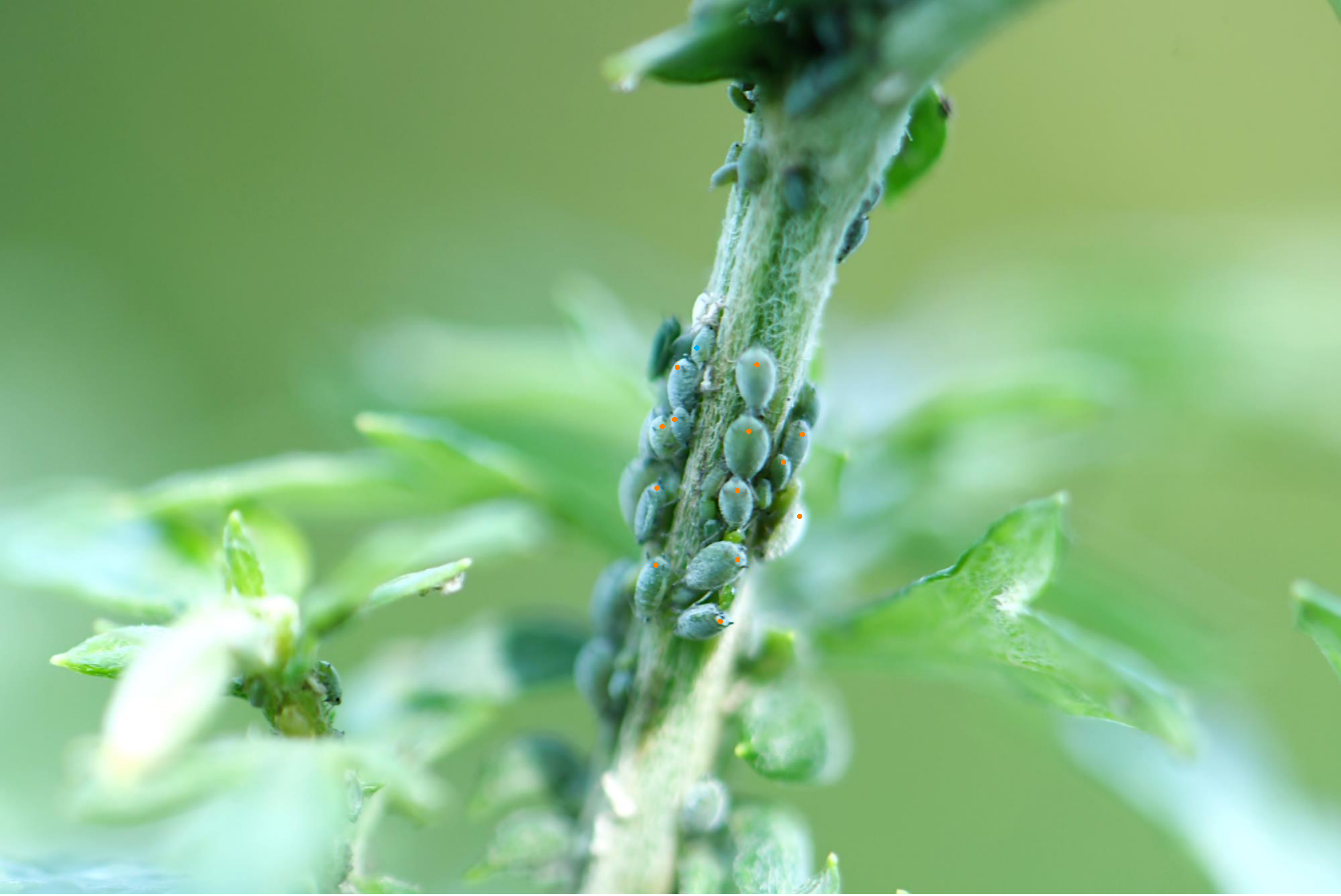

Picture ID: 34, *Ap. kurosawai* on *Ar. indica*, 20 October 2024.

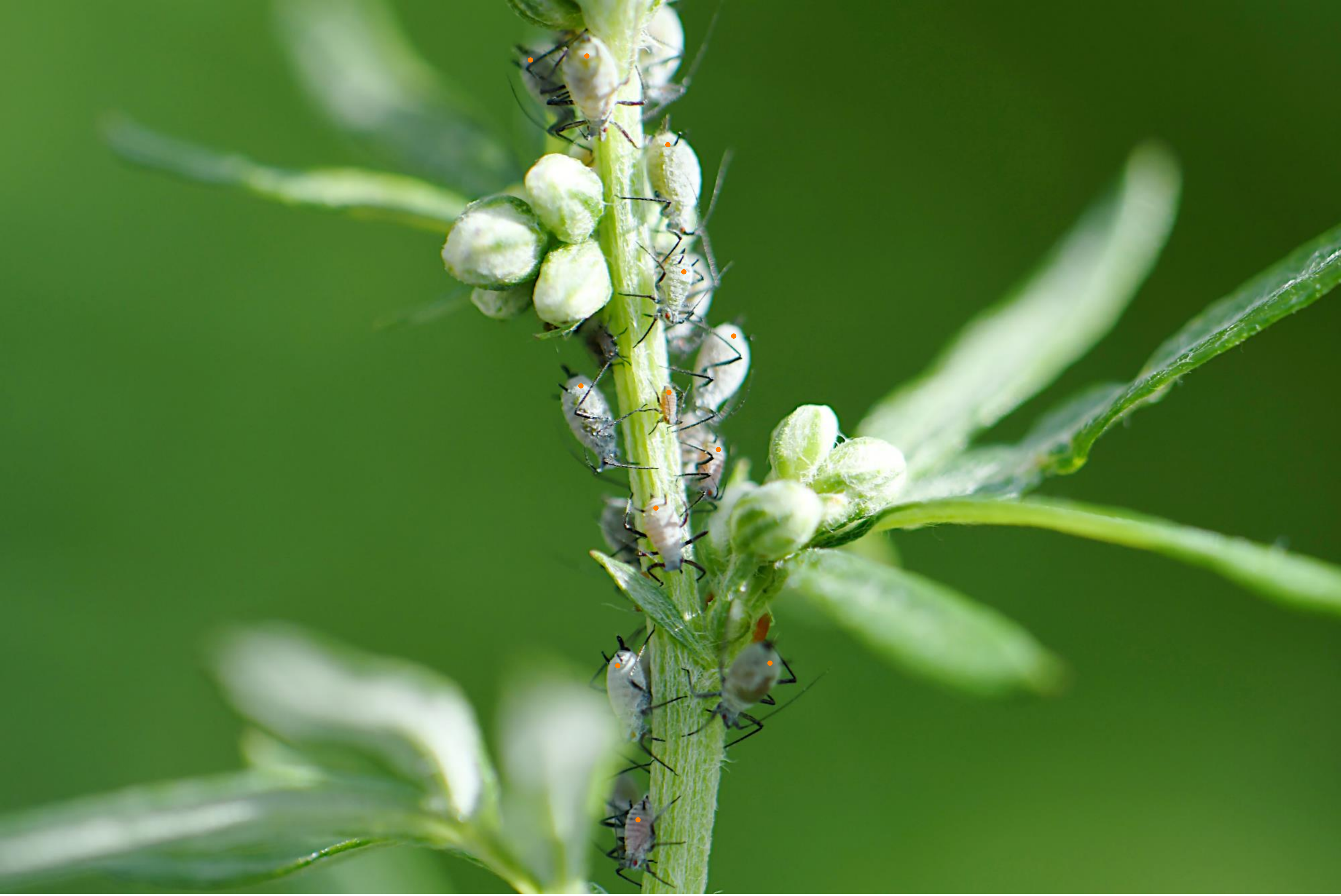

Picture ID: 35, *Macrosiphoniella yomogifoliae* on *A. indica*, 20 October 2024.

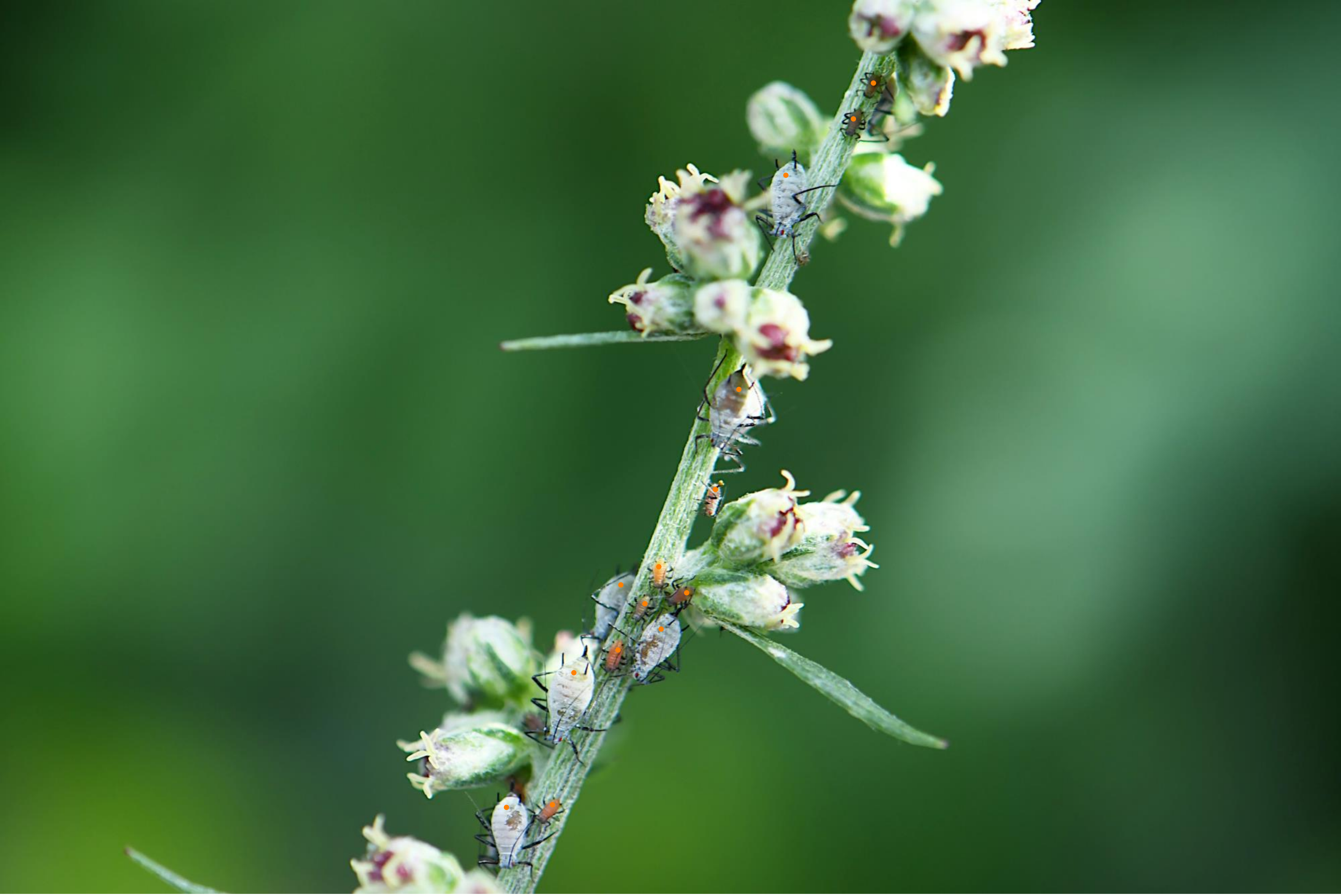

Picture ID: 36, *Ma. yomogifoliae* on *Ar. indica*, 20 October 2024.

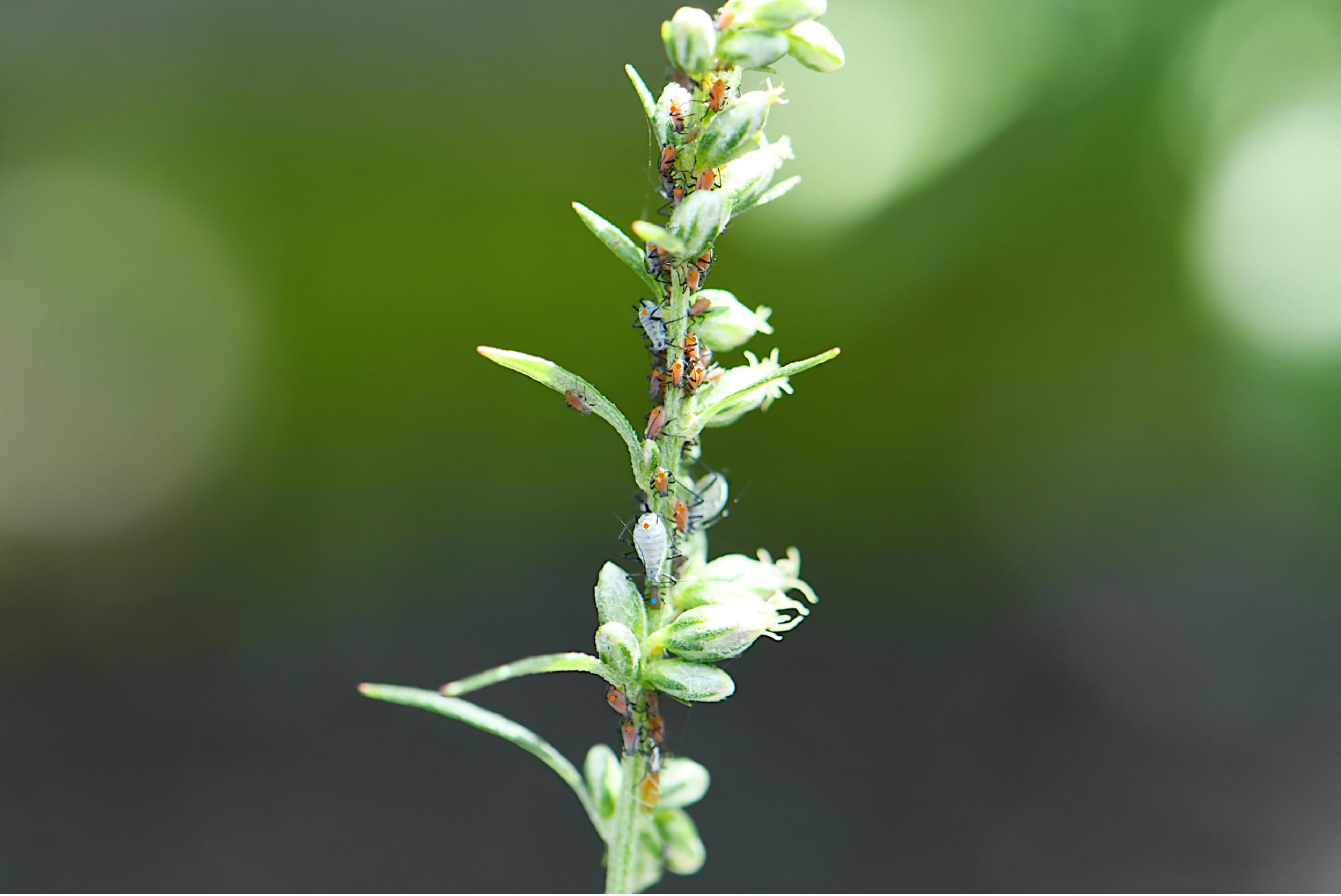

Picture ID: 37, *Ma. yomogifoliae* on *Ar. indica*, 20 October 2024.

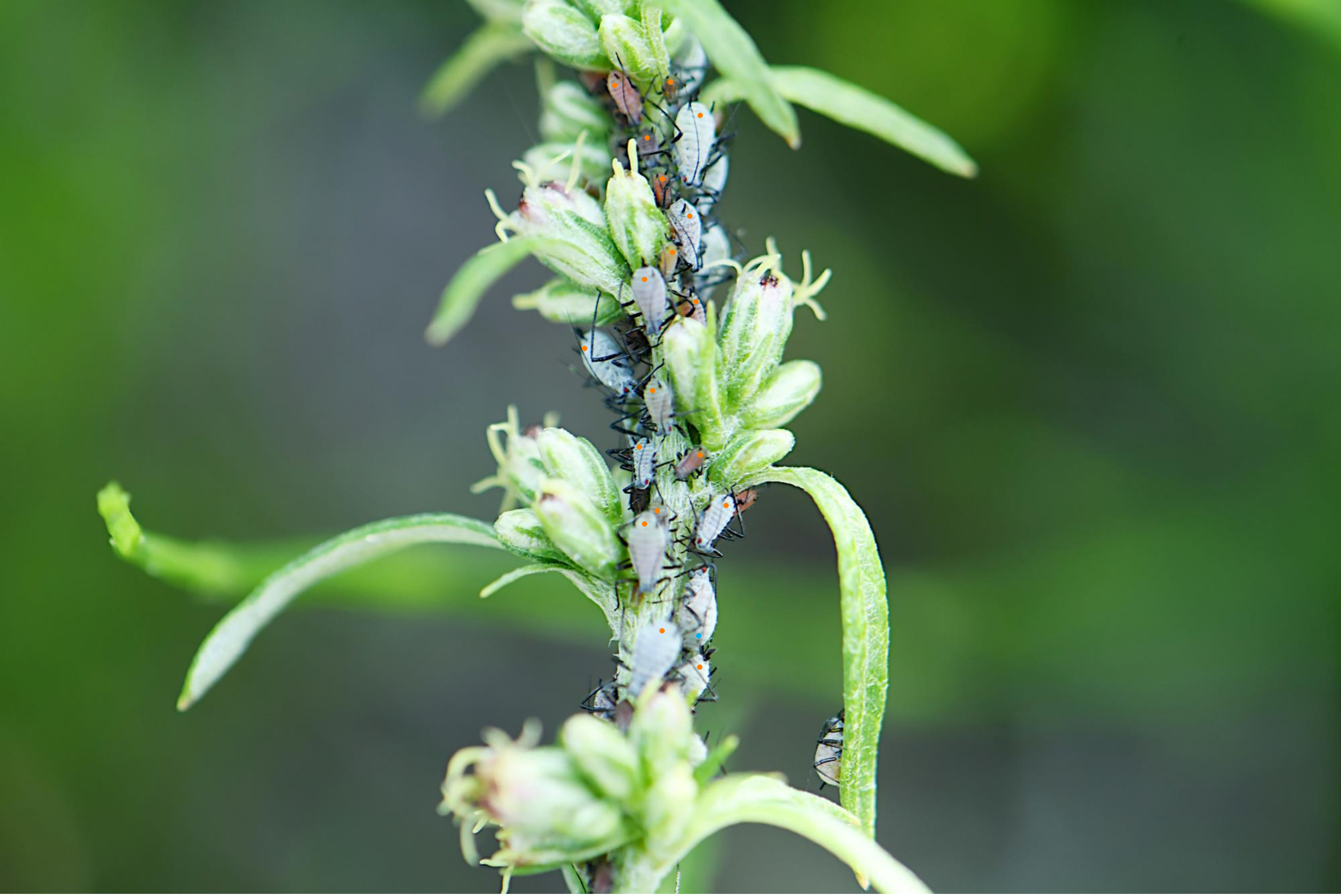

Picture ID: 38, *Ma. yomogifoliae* on *Ar. indica*, 20 October 2024.

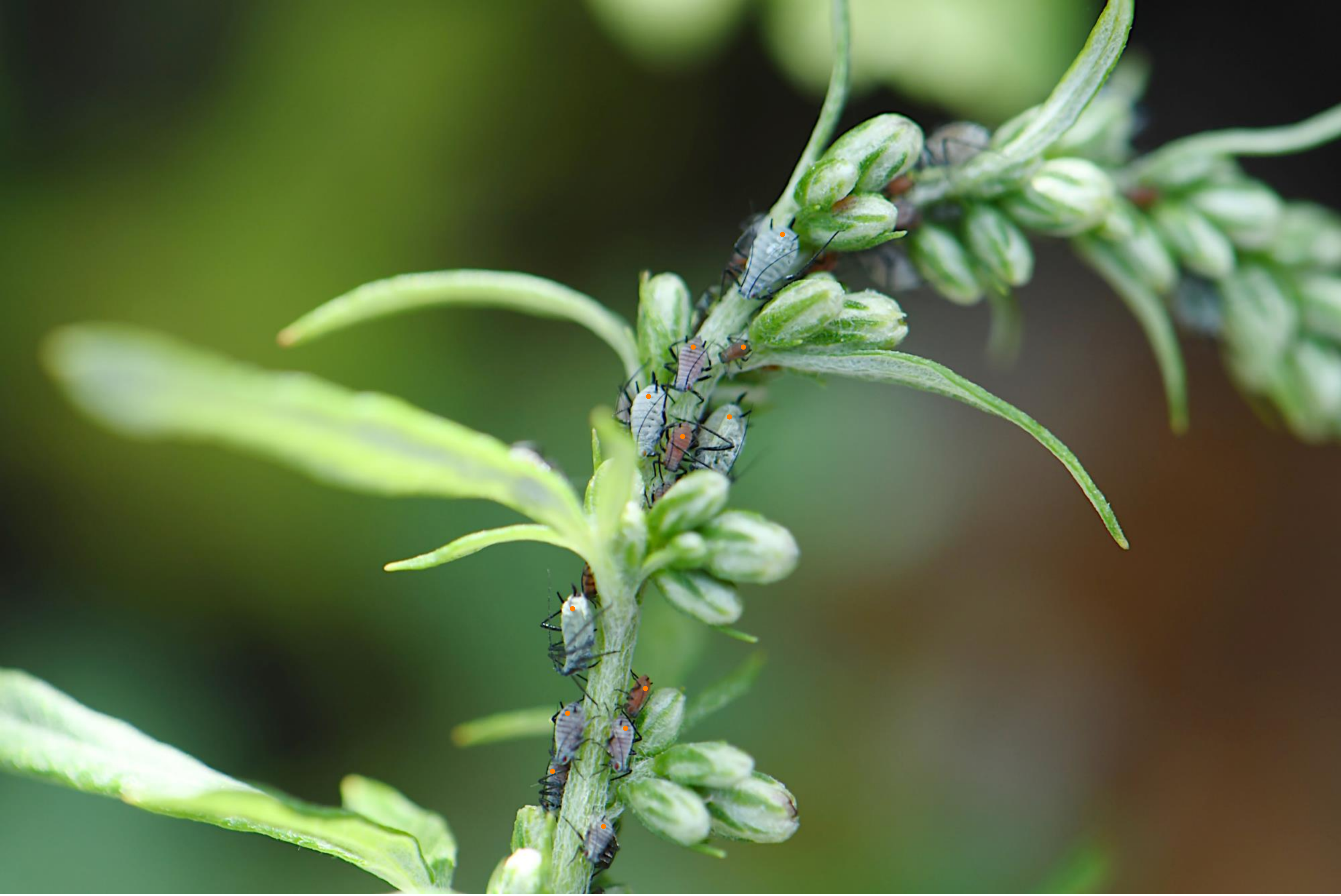

Picture ID: 39, *Ma. yomogifoliae* on *Ar. indica*, 20 October 2024.

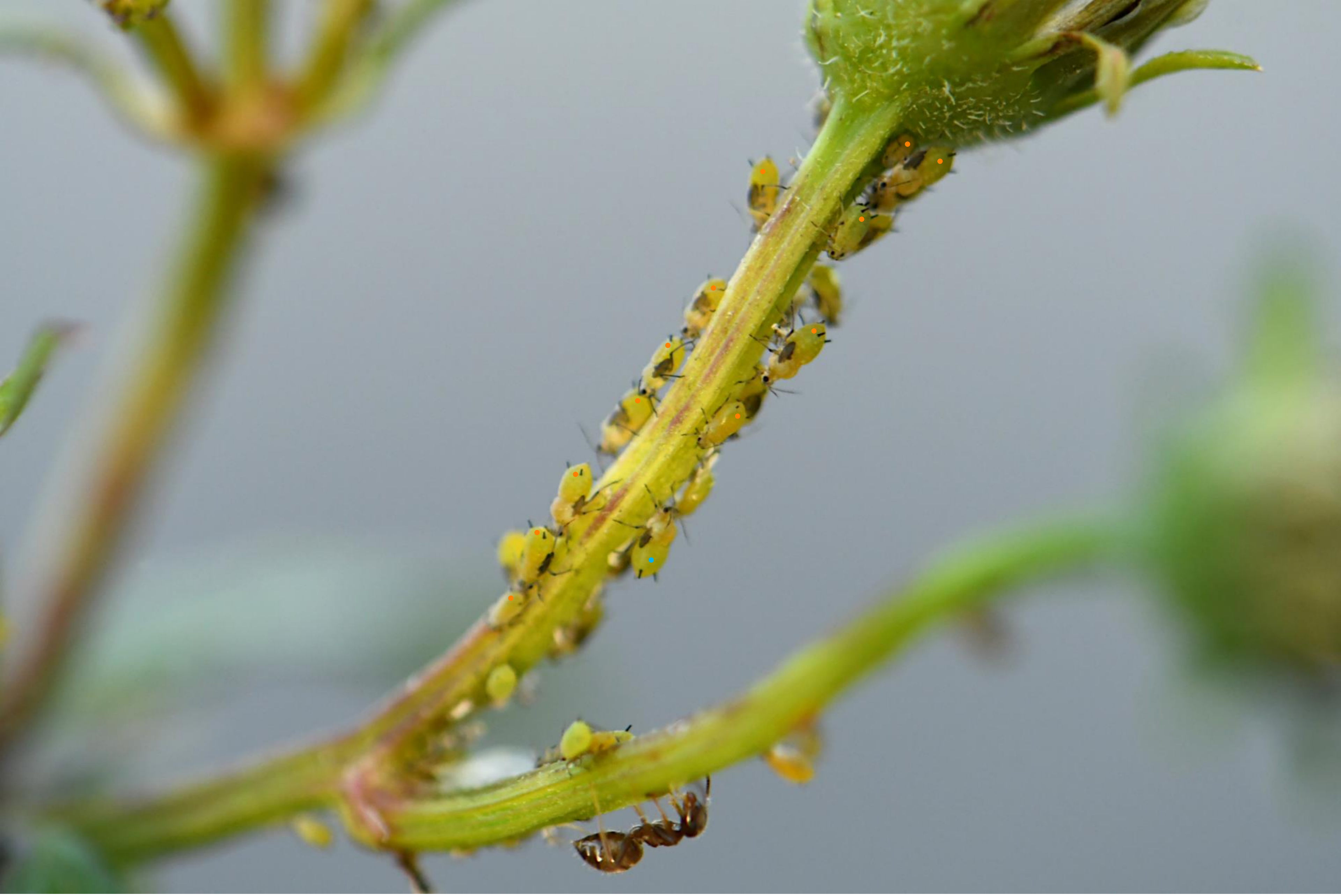

Picture ID: 40, *Aphis spiraecola* on *Bidens pilosa*, 8 November 2024.

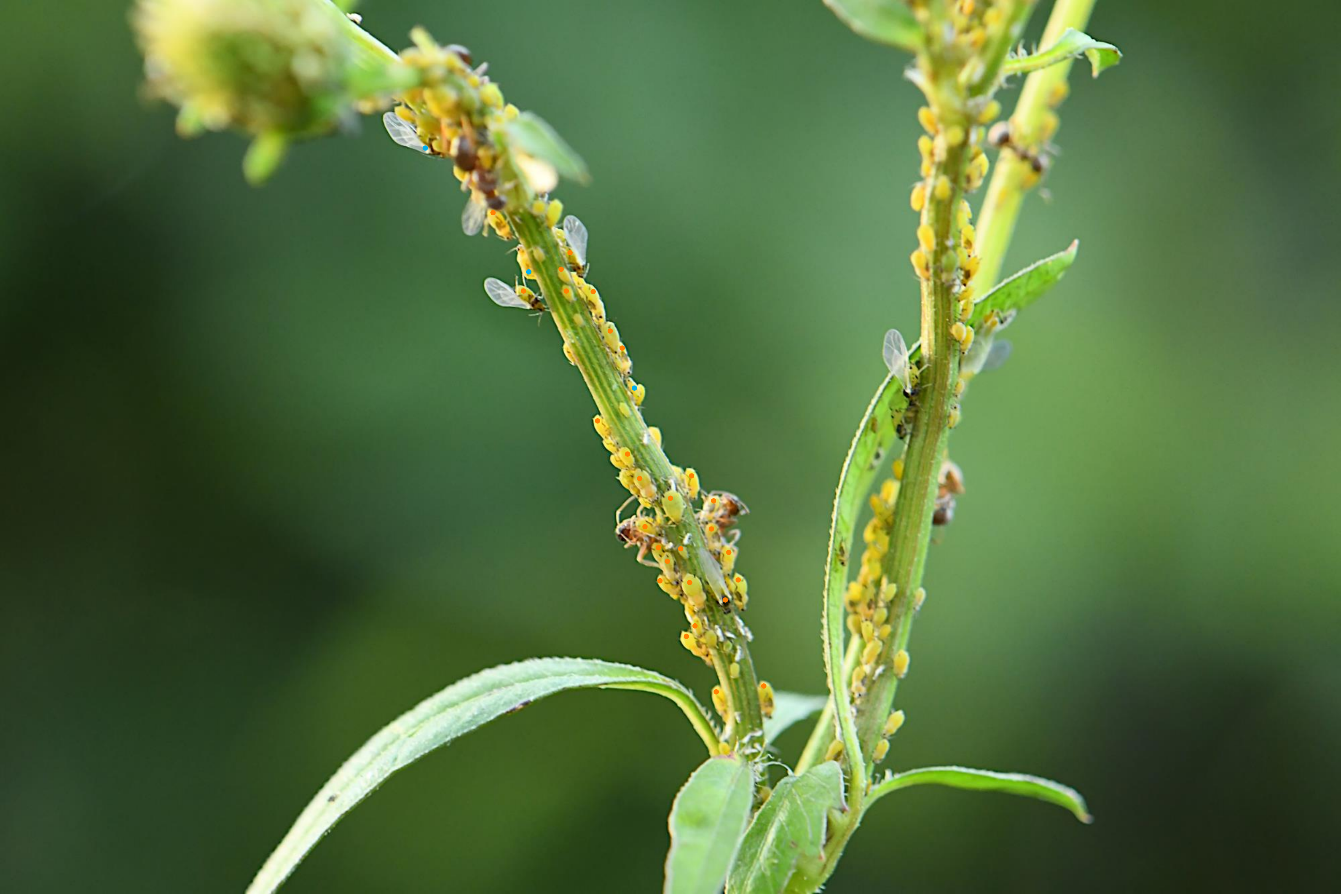

Picture ID: 41, *Ap. spiraecola* on *Bi. pilosa*, 8 November 2024.

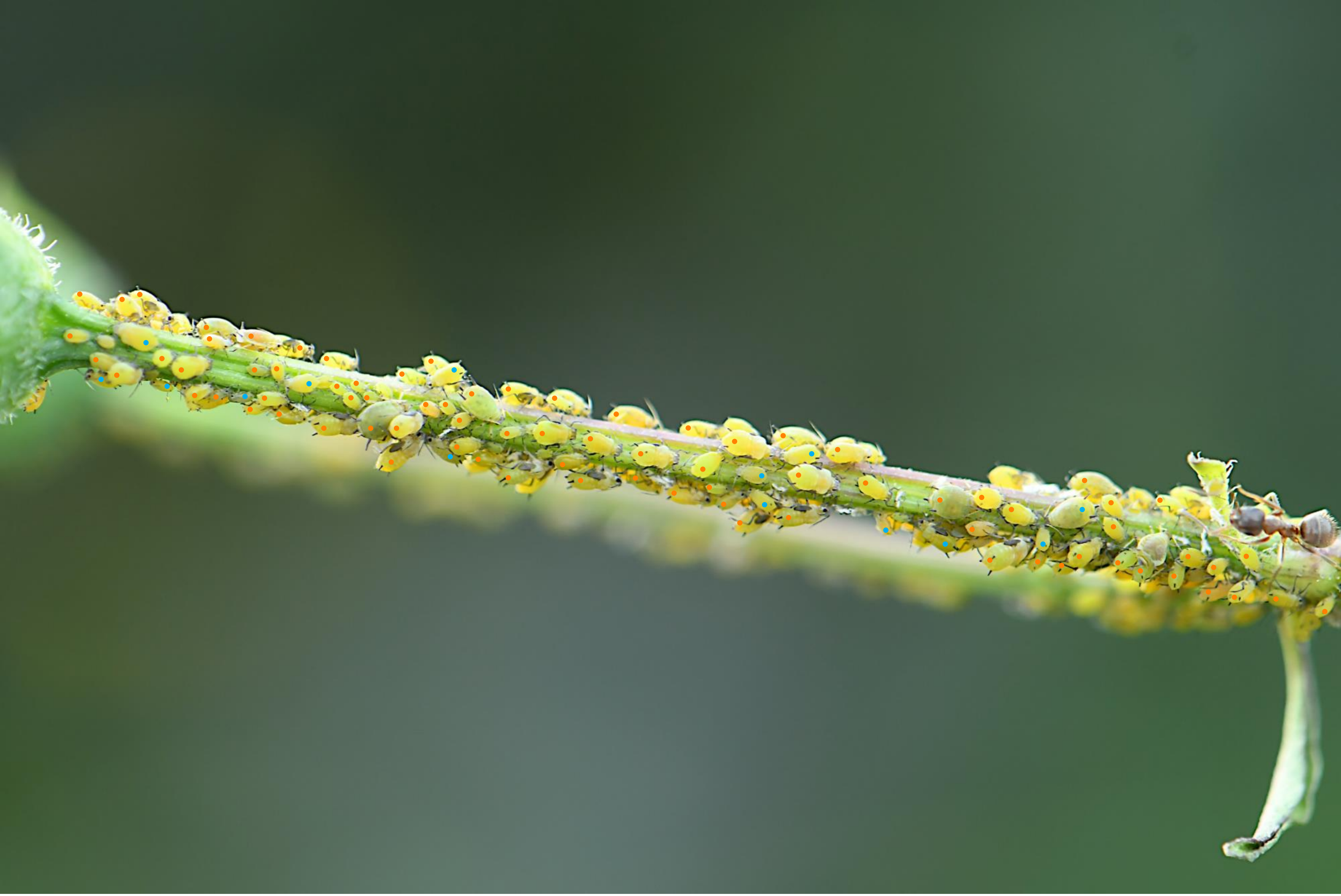

Picture ID: 42, *Ap. spiraecola* on *Bi. pilosa*, 8 November 2024.

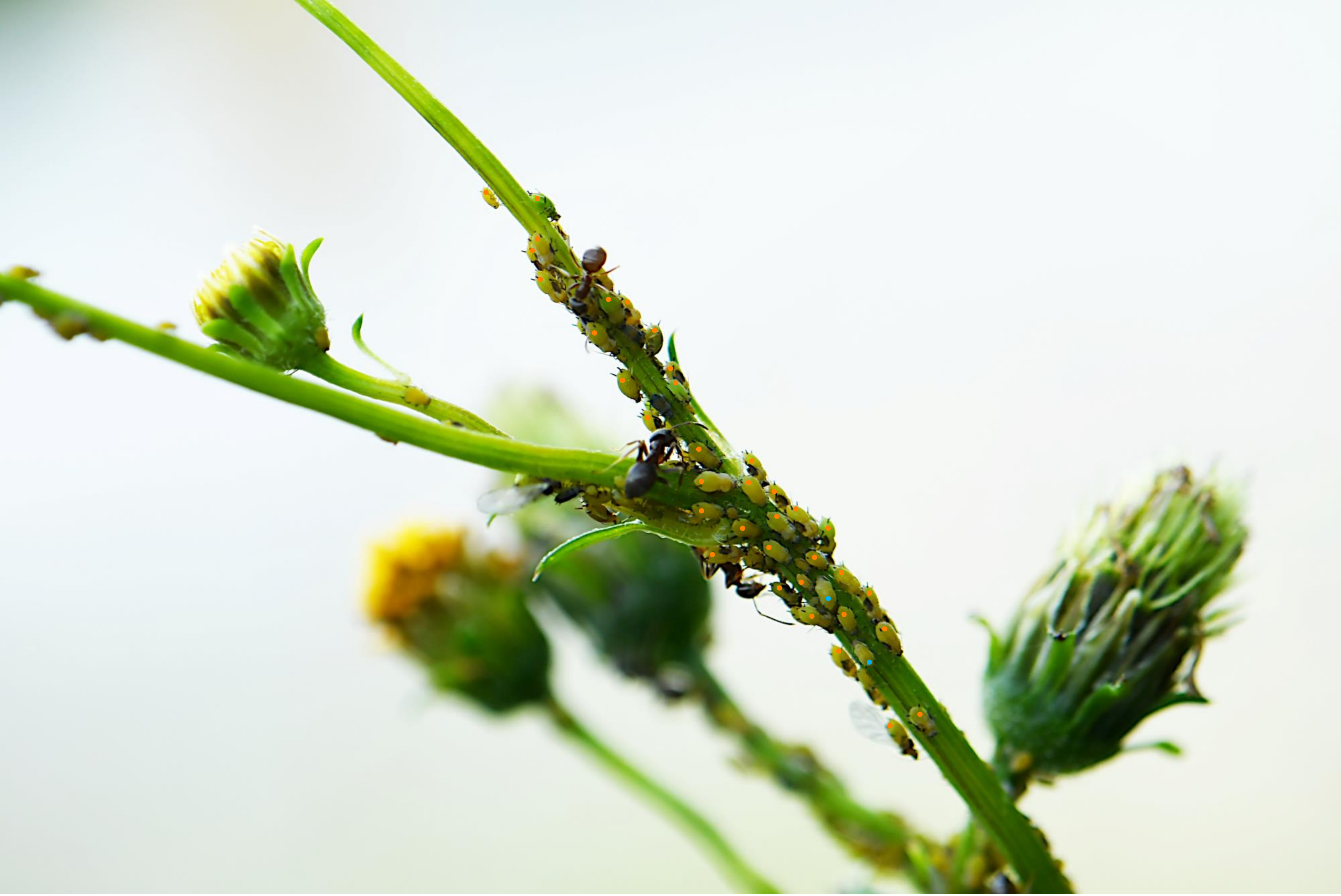

Picture ID: 43, *Ap. spiraecola* on *Bi. pilosa*, 8 November 2024.

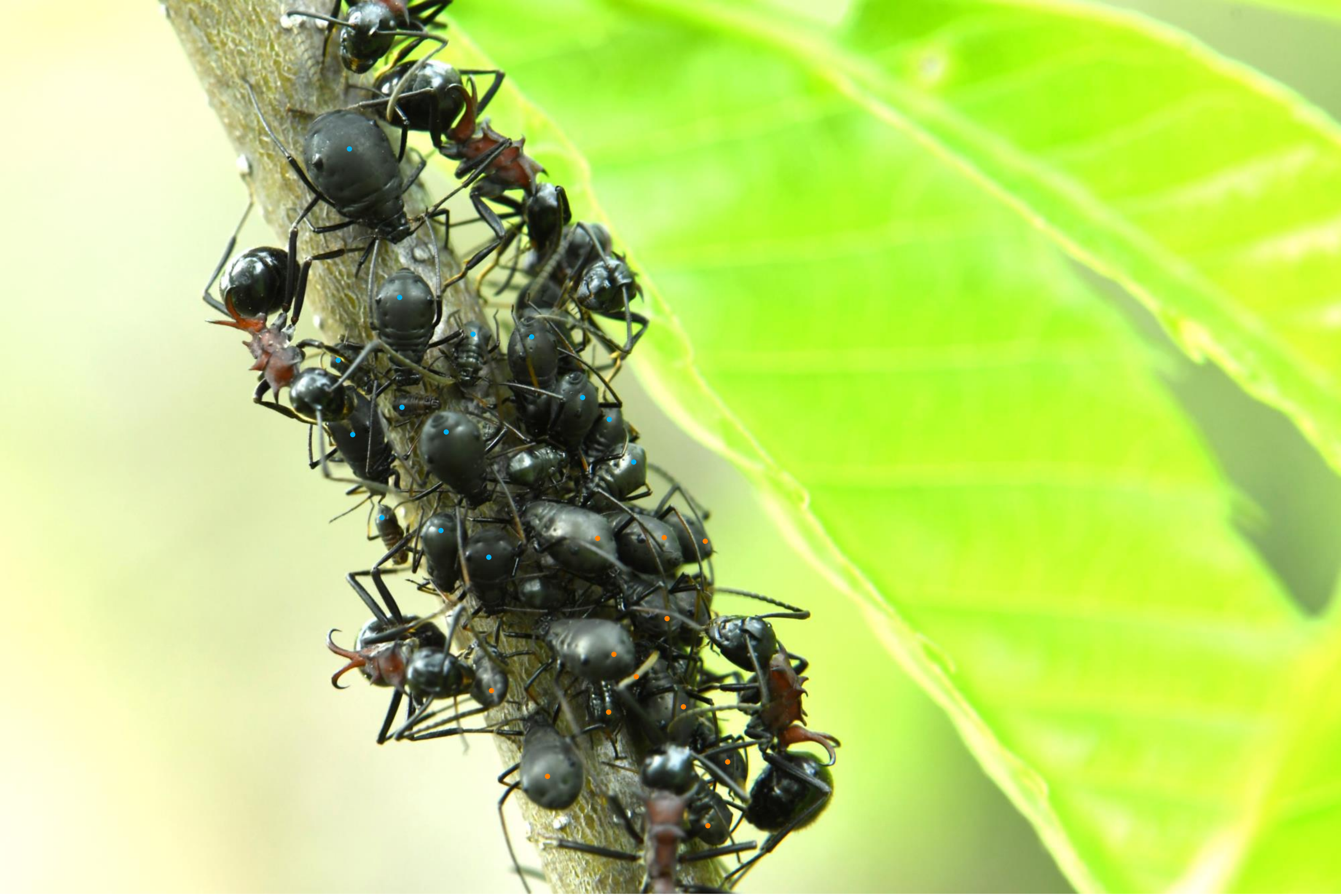

Picture ID: 44, *Lachnus tropicalis* on *Castanea crenata*, 17 April 2024.

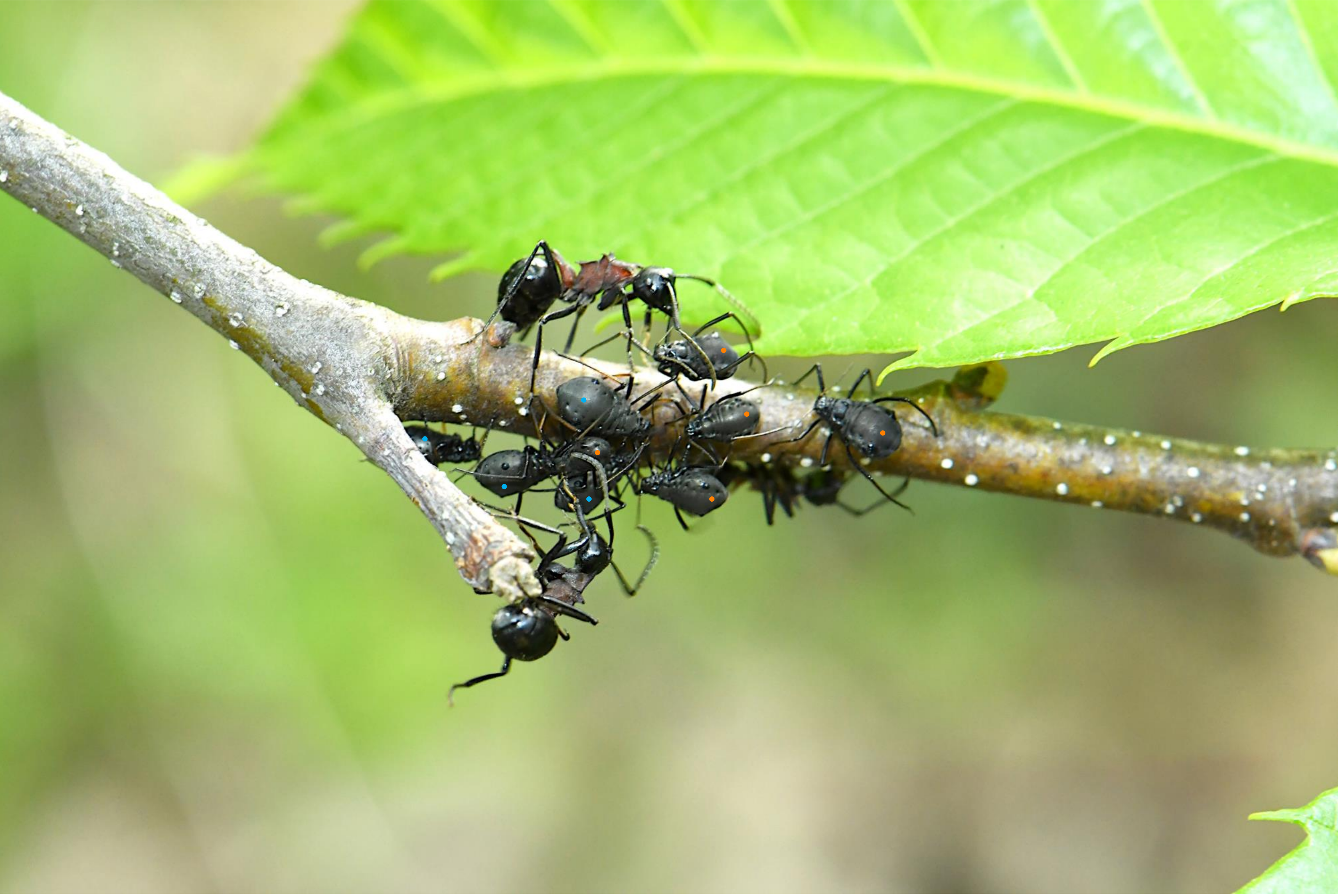

Picture ID: 45, *L. tropicalis* on *Ca. crenata*, 19 April 2024.

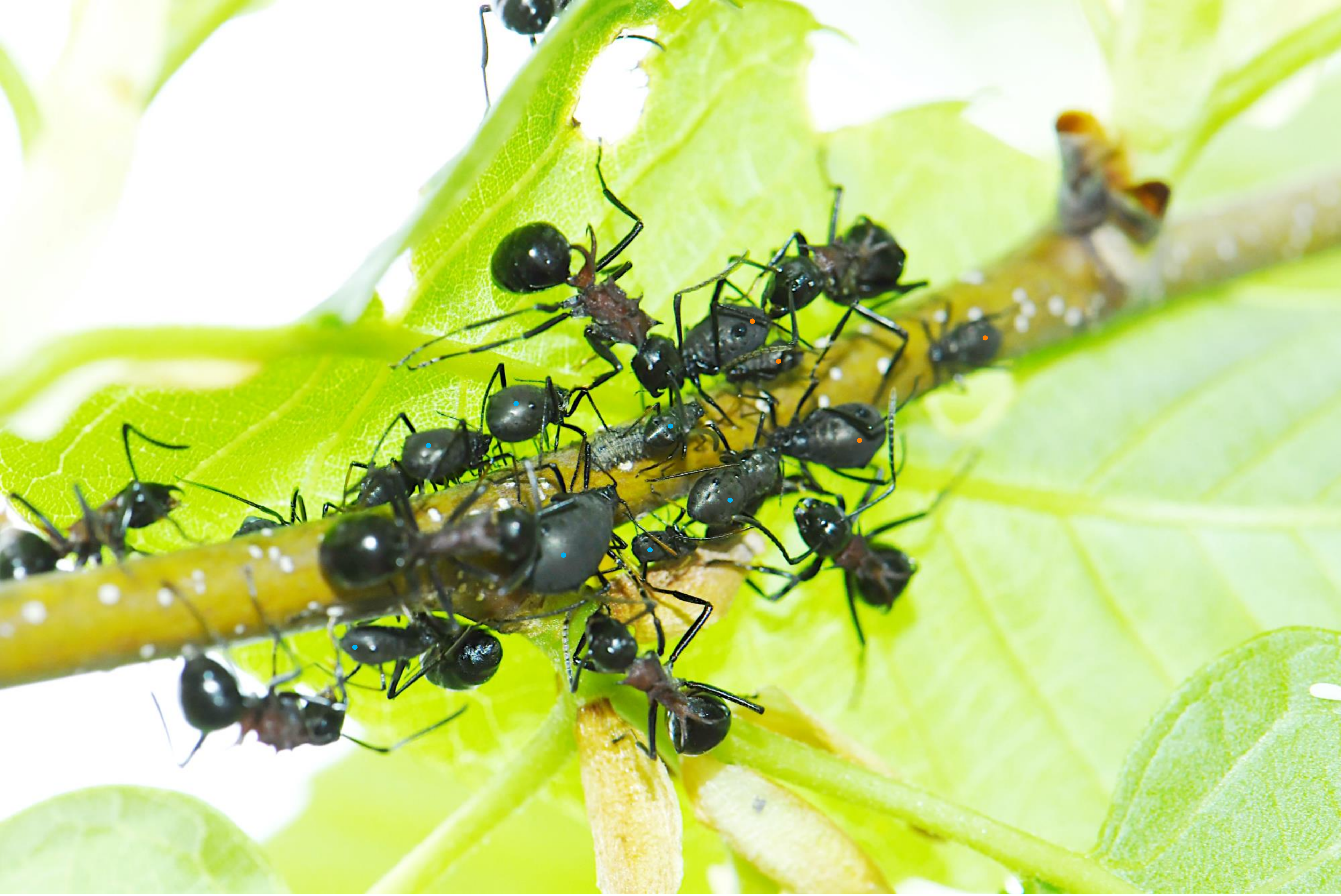

Picture ID: 46, *L. tropicalis* on *Ca. crenata*, 19 April 2024.
